# The Borel map in locally integrable structures

**DOI:** 10.1007/s00208-019-01811-w

**Published:** 2019-02-15

**Authors:** Giuseppe Della Sala, Paulo D. Cordaro, Bernhard Lamel

**Affiliations:** 1 American University of Beirut, Beirut, Lebanon; 2 University of Sao Paulo, São Paulo, Brazil; 3 University of Vienna, Vienna, Austria

**Keywords:** ., Primary: 35N10

## Abstract

Given a locally integrable structure ${\mathcal {V}}$ over a smooth manifold $\varOmega$ and given $p\in \varOmega$ we define the * Borel map of*${\mathcal {V}}$* atp* as the map which assigns to the germ of a smooth solution of ${\mathcal {V}}$ at *p* its formal Taylor power series at *p*. In this work we continue the study initiated in Barostichi et al. (Math. Nachr. 286(14–15):1439–1451, 2013), Della Sala and Lamel (Int J Math 24(11):1350091, 2013) and present new results regarding the Borel map. We prove a general necessary condition for the surjectivity of the Borel map to hold and also, after developing some new devices, we study some classes of CR structures for which its surjectivity is valid. In the final sections we show how the Borel map can be applied to the study of the algebra of germs of solutions of ${\mathcal {V}}$ at *p*.

## Introduction

The purpose of this paper is to discuss our recent results on the *Borel map* and the *Borel property* for locally integrable structures. If one thinks about an integrable structure as a system of (linear, first order) PDEs with the right number of basic solutions, it becomes an intriguing question to study the relationship between *formal solutions* (i.e. formal power series in the solutions of the structure) and *solutions*. The relationship between the two comes, of course, from associating to a smooth solution its formal Taylor series at a distinguished point (e.g. the origin) in the structure. The Taylor series of a solution can be written as a series in the elements of a set of basic first integrals $\{ Z_1, \dots , Z_m \}$ defined near the origin; we refer to this map, defined by

$$\begin{aligned} {\mathfrak {b}}:{\mathfrak {S}}_0 \rightarrow {{\mathbb {C}}}\llbracket Z_1, \dots , Z_m \rrbracket , \quad \mathfrak {b}(u) = \sum _{\alpha \in {\mathbb {N}}^m} u_\alpha Z^\alpha , \end{aligned}$$

where the $u_\alpha$ are appropriate derivatives evaluated at 0 of *u*, as the *Borel map* (at the origin).

We have started the study of this map, in particular the natural question of when it is surjective (the *Borel property*), in a series of papers of the second author with Barostichi and Petronilho [3] and of the first and the third author in the context of CR structures [6]. In our current paper, we can give important insights into the nature of the geometric properties of the structure determining whether the Borel property holds or not, and we find relationships with interesting open questions in the analysis of locally integrable structures.

Before we begin with the discussion of our results, we refer the reader to Sect. 2 for thorough definitions of locally integrable structures, the Borel map (which associates to any smooth solution its formal solution), and the Borel property (meaning that the Borel map is surjective). The Borel property can be used to understand, and, in some circumstances, bridge the gap between the local algebra of power series spaces and the analysis of properties of smooth solutions.

In Sect. 3 we use functional analytic methods in order to characterize (abstractly) when the Borel property holds in Proposition 3.2: roughly stated, the Borel property holds if and only if the following is true: when one can uniformly control the action of a sequence of differential operators on the solutions of the structure by the $C^\ell$-norm on some compact set, then the operators in the sequence need to be of bounded order. We use this fact to provide some conceptually simpler and, in view of later results, cleaner proofs of the fact that the existence of peak functions of finite type (or in the locally integrable case, the fact that property ($\mathfrak {B}$) holds) is sufficient for the Borel property to hold, and for the fact that the existence of a flat solution is necessary for the Borel property to hold.

However, the results in our current paper show that geometric properties of this form are far too rough to understand the Borel map. We hope that this means that understanding the Borel map is more feasible than understanding whether e.g. a peak function exists (which is a very hard undertaking, see e.g. the survey by Noell [11]), as it turns out that the Borel map is a very subtle instrument which feels a lot of the intrinsic geometry of the integrable structure. In particular, the present results give hope (and lead to some actual conjectures) that one can reach a satisfactory geometric characterization of the Borel property, and show that its application to e.g. the structure of ideals of solutions gives important insights into the behaviour of solutions.

There are also structural aspects of the Borel map which make its study very appealing: We encounter one such aspect when we study *partial Borel maps* in Sect. 4. Partial Borel maps are defined as restrictions of the Borel map to solutions which are flat in a number of the basic solutions, giving rise to formal series only depending on the other basic solutions. It turns out (Theorem 4.1) that the Borel map is surjective if and only if the partial Borel maps associated to a choice of a set of basic solutions and to its complementary set are both surjective.

Our main new necessary condition (Theorem 6.1) for the surjectivity of the Borel map is that the polynomial hull of *Z*(*K*), where $Z = (Z_1, \dots , Z_m)$ is the embedding of the structure into ${{\mathbb {C}}}^m$ by means of a set of basic solutions, does not contain any analytic discs. It is tempting to conjecture (especially when considering the proof of that statement) that this condition is not only necessary but also sufficient.

Hence one of the remaining objectives of the paper is a discussion of the possible gap between the necessity of the condition and the stronger conditions known to be sufficient. A particular case in question is an application of the result on partial Borel maps to structures whose characteristic set is of maximal dimension; in that case, we see that the Borel map is surjective if none of the solutions of the structure is open (Theorem 7.3).

This result highlights yet another interesting problem to which the Borel property has a curious connection, namely the question whether there is a solution (with nontrivial differential in a noncharacteristic direction) which is actually open; it also, therefore connects with the question of whether a maximum principle is valid for solutions of the given structure. We shall, however, in this paper not follow these lines of inquiry further.

Instead, we have decided to focus on the study of what we think is the main geometric question left over in our approach here in a special model case of tube structures. We obtain a rather complete picture in that case, which is discussed in Sect. 8. We show in Theorem 8.1 that if neither the known condition for surjectivity (property $(\mathfrak {B})$), nor the condition for failure of surjectivity (open mapping property) hold, that we can reduce the problem to studying sets which are in some sense “characteristic” for property $\mathfrak {B}$. and it is in many cases the geometry of these sets which allows us to determine whether the Borel map is onto or not (Theorems 8.2 and 8.3).

In the last two sections of the paper, Sects. 9 and 10, we study two particular algebraic aspects of the ring of solutions: we first show that its maximal ideal is finitely generated by a set of basic solutions if property $(\mathfrak {B})$ holds (Theorem 9.1). There are also other situations in which we can guarantee this basic property, but we would definitely like to know whether the maximal ideal in the ring of solutions is always generated by a set of basic solutions (or not). In the other extreme, we also show that principal manifold ideals automatically (without further assumptions on the structure) satisfy the Nullstellensatz (Lemma 10.1).

We would like to note that the current paper leaves open a number of fascinating problems concerning the behaviour of the Borel map and the relation between the algebra of formal solutions and the algebra of solutions; we discuss a number of them in section 11.

## The Borel property in locally integrable structures

**A.** Let $\varOmega$ be a smooth (paracompact) manifold of dimension *N* over which we assume given a locally integrable structure ${\mathcal {V}}$ of rank *n*. Thus ${\mathcal {V}}$ is a vector subbundle of ${{\mathbb {C}}}{\mathrm {T}}\varOmega$ of rank *n* whose orthogonal bundle ${\mathcal {V}}^\perp \subset {{\mathbb {C}}}{\mathrm {T}}^*\varOmega$ is locally spanned by the differentials of $m=N-n$ smooth functions.

If $p\in \varOmega$ we set

$$\begin{aligned} {\mathfrak {S}}_p \doteq \{f\in C^\infty _p:\, {\mathrm {L}}f=0, \,\, \forall \hbox { sections } {\mathrm {L}}\hbox { of } {\mathcal {V}}\hbox { near } p\}, \end{aligned}$$

where we are denoting by $C^\infty _p$ the ring of germs of smooth functions at *p*. It is clear that ${\mathfrak {S}}_p$ is also a ring.

For each $k\ge 0$ let ${\mathfrak {m}}_p^k$ denote the ideal of $C^\infty _p$ formed by all $f\in C^\infty _p$ for which there is a constant $C>0$ such that $|f(q)|\le Cd(q,p)^{k+1}$ for *q* in a neighborhood of *p*.^1^ It is also clear that ${\mathfrak {m}}_p^{k+1}\subset {\mathfrak {m}}_p^k$ for every $k\ge 0$ and that ${\mathfrak {m}}_p^k\cap {\mathfrak {S}}_p$ is an ideal of ${\mathfrak {S}}_p$. We can then form the quotient ring ${\mathcal {J}}({\mathcal {V}})^k_p \doteq {\mathfrak {S}}_p/({\mathfrak {m}}_p^k\cap {\mathfrak {S}}_p)$, which is called the * ring ofk*-* jets of solutions atp*. We have well defined homomorphisms $\iota _k:{\mathcal {J}}({\mathcal {V}})^k_p\rightarrow {\mathcal {J}}({\mathcal {V}})^{k-1}_p$, $k\ge 1$, induced by the inclusions ${\mathfrak {m}}_p^{k}\subset {\mathfrak {m}}_p^{k-1}$. Furthermore $\iota _k\circ \pi _k= \pi _{k-1}$, $k\ge 1$, where $\pi _k$ stands for the quotient map ${\mathfrak {S}}_p\rightarrow {\mathcal {J}}({\mathcal {V}})_p^k$. We can form the projective limit

$$\begin{aligned} {\mathcal {J}}({\mathcal {V}})^\infty _p\doteq \lim _{\leftarrow } {\mathcal {J}}({\mathcal {V}})^k_p \end{aligned}$$

which is then called the ring of * formal solutions for*${\mathcal {V}}$* atp*. Recall that ${\mathcal {J}}({\mathcal {V}})^\infty _p$ is the set of all sequences $(s_k)_{k\ge 0}$ with $s_k\in {\mathcal {J}}^k_p$ and $s_{k-1}= \iota _k(s_k)$ for every $k\ge 1$. Finally we define

$$\begin{aligned} {\mathfrak {b}}_{{\mathcal {V}},p}:{\mathfrak {S}}_p\rightarrow {\mathcal {J}}({\mathcal {V}})^\infty _p, \quad {\mathfrak {b}}_{{\mathcal {V}},p}(u)= (\pi _k(u))_{k\ge 0}\, \end{aligned}$$

### Definition 2.1

We shall refer to the ring homomorphism ${\mathfrak {b}}_{{\mathcal {V}},p}$ as the Borel map for ${\mathcal {V}}$ at *p*. We shall also say that ${\mathcal {V}}$ satisfies the Borel property at *p* if ${\mathfrak {b}}_{{\mathcal {V}},p}$ is surjective.

**B.** Let $${\mathcal {V}}$$ be a smooth, locally integrable structure defined on a smooth manifold $$\varOmega $$ and let $$p\in \varOmega $$. According to [4] we can assert the following: *p* is the center of a smooth coordinate system $$(x_1,\ldots ,x_m,$$$$t_1,\ldots ,t_n)$$, which can be assumed defined in a product $$U=B\times \varTheta $$, where *B* (respectively $$\varTheta $$) is an open ball centered at the origin in $${{\mathbb {R}}}_x^m$$ (respectively $${{\mathbb {R}}}_t^n$$), over which there is defined a smooth, real vector-valued function $$\varPhi (x,t)=(\varPhi _1(x,t),\dots ,\varPhi _m(x,t))$$ satisfying $$\varPhi (0,0)=0$$, $$D_x\varPhi (0,0)=0$$, such that the differential of the functions

$$\begin{aligned} Z_k(x,t)=x_k+i\varPhi _k(x,t),\qquad k=1,\ldots , m, \end{aligned}$$

span $${\mathcal {V}}^\perp $$ over *U*.

Moreover $\hbox {d}Z_1,\ldots , \hbox {d}Z_m, \hbox {d}t_1,\ldots ,\hbox {d}t_n$ span ${{\mathbb {C}}}{\mathrm {T}}^*\varOmega$ over *U*.

Over *U* we can define smooth vector fields

$$\begin{aligned} {\mathrm {M}}_k=\sum _{k'=1}^m\mu _{kk'}(x,t)\frac{\partial }{\partial x_{k'}}, \quad k=1,\dots ,m \end{aligned}$$

characterized by the rule

$$\begin{aligned} {\mathrm {M}}_kZ_{k'}=\delta _{k,k'}\,, \quad k,k'=1,\ldots , m. \end{aligned}$$

It follows that the complex vector fields

$$\begin{aligned} {\mathrm {L}}_j=\frac{\partial }{\partial t_j}-i\sum _{k=1}^m\frac{\partial \varPhi _k}{\partial t_j}(x,t){\mathrm {M}}_k, \quad j=1,\dots ,n, \end{aligned}$$

span ${\mathcal {V}}|_U$. Moreover, ${\mathrm {L}}_1,\ldots ,{\mathrm {L}}_n,{\mathrm {M}}_1,\ldots ,{\mathrm {M}}_m$ span ${{\mathbb {C}}}{\mathrm {T}}\varOmega |_U$.

The following relations are easily checked, for every $j,j'=1,\ldots , n$, $k,k'=1,\ldots ,m$:

$$\begin{aligned} \hbox {d}Z_k({\mathrm {L}}_j)=0,\,\, \hbox {d}Z_k({\mathrm {M}}_{k'})=\delta _{kk'},\,\,\, \hbox {d}t_j({\mathrm {L}}_{j'})=\delta _{jj'},\,\,\, \hbox {d}t_j({\mathrm {M}}_k)=0, \end{aligned}$$

from which we conclude that ${\mathrm {L}}_1,\ldots ,{\mathrm {L}}_n,{\mathrm {M}}_1,\ldots ,{\mathrm {M}}_m$ are pairwise commuting.

Set, for $W\subset U$ open,

$$\begin{aligned} {\mathfrak {S}}(W)\doteq \{ u\in C^\infty (W): {\mathrm {L}}_ju=0, \quad j=1,\ldots ,n\}; \end{aligned}$$

it follows, according to the previously established, that

$$\begin{aligned} {\mathfrak {S}}_0 = \lim _{W\rightarrow \{0\}} {\mathfrak {S}}(W). \end{aligned}$$

We are now ready to give a concrete representation of the Borel map for ${\mathcal {V}}$ at the origin using this basic set of generators $\{Z_1,\ldots ,Z_m\}$. Firstly we observe that if $u\in {\mathfrak {S}}_0$ then all derivatives up to order *k* of the solution

$$\begin{aligned} v_k \doteq u - \sum _{|\alpha |\le k} \frac{{\mathrm {M}}^\alpha u(0)}{\alpha !} Z(x,t)^\alpha \end{aligned}$$

vanish at the origin; this can be easily seen for $({\mathrm {M}}^\beta {\mathrm {L}}^\gamma v_k)(0)=0$ if $\beta \in {\mathbb {Z}}^m_+$, $\gamma \in {\mathbb {Z}}^n_+$, $|\beta |+|\gamma |\le k$. In particular $v_k\in {\mathfrak {m}}_0^k \cap {\mathfrak {S}}_0$ and hence the class of *u* in ${\mathcal {J}}({\mathcal {V}})^k_0$ equals that of $u-v_k$, which gives rise to an isomorphism

$$\begin{aligned} \eta _k:{\mathcal {J}}_0^k({\mathcal {V}})_0 \longrightarrow {{\mathbb {C}}}_{k}[Z_1,\ldots , Z_m] \end{aligned}$$

where the latter denotes the vector space of all polynomials in $Z_1,\ldots ,Z_m$ of order $\le k$. Furthermore, for each $k\ge 1$ we have commutative diagrams 
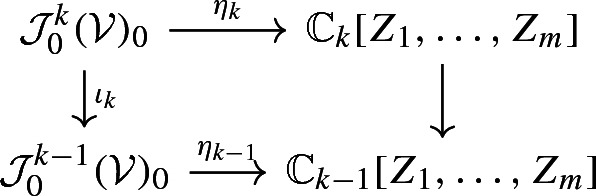
 where the vertical arrows at the right stand for the natural projections. If we recall that the ring of formal power series ${{\mathbb {C}}}\llbracket Z_1,\ldots ,Z_m \rrbracket$ equals the projective limit $\lim _{\leftarrow }{{\mathbb {C}}}_{k}[Z_1,\ldots , Z_m]$ we finally obtain an isomorphism

$$\begin{aligned} \eta : {\mathcal {J}}_0^\infty \longrightarrow {{\mathbb {C}}}\llbracket Z_1,\ldots , Z_m \rrbracket . \end{aligned}$$

For the representation of the Borel map for ${\mathcal {V}}$ at the origin in terms of $\{Z_1,\ldots ,Z_m\}$ we must just observe that the map ${\mathfrak {b}}: {\mathfrak {S}}_0 \longrightarrow {{\mathbb {C}}}\llbracket Z_1,\dots ,Z_m \rrbracket$ given by

$$\begin{aligned} {\mathfrak {b}}(u) = \sum _{\alpha \in {\mathbb {Z}}^m}\frac{({\mathrm {M}}^\alpha u)(0)}{\alpha !}Z(x,t)^\alpha \end{aligned}$$

makes the diagram 
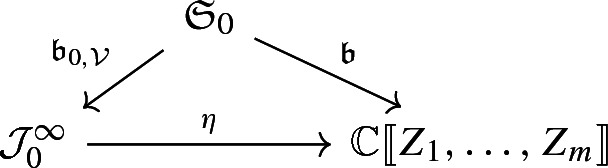
 commutative. In particular we conclude that the Borel property for ${\mathcal {V}}$ holds at the origin if and only if ${\mathfrak {b}}$ is surjective. Moreover the image of ${\mathfrak {b}}_{0,{\mathcal {V}}}$ and ${\mathfrak {b}}$ are isomorphic.

## General properties of the Borel map

**A.** It is our goal in this work to study not only conditions to ensure the surjectivity of ${\mathfrak {b}}$ but also to analyze its algebraic properties and apply them to the study of the properties of the algebra ${\mathfrak {S}}_0$.

We first recall a result proved in [3], Lemma 3.2: ${\mathfrak {b}}$ is surjective if and only if there exists an open neighborhood of the origin $V\subset U$ such that

1 $$\begin{aligned} {\mathfrak {b}}_V : {\mathfrak {S}}(V) \longrightarrow {{\mathbb {C}}}\llbracket Z_1,\ldots ,Z_m \rrbracket \end{aligned}$$

is surjective. Here ${\mathfrak {b}}_V= {\mathfrak {b}}\circ \sigma _V$, where $\sigma _V: {\mathfrak {S}}(V)\rightarrow {\mathfrak {S}}_0$ asssociates to $u\in {\mathfrak {S}}(V)$ its germ at the origin.

Both ${\mathfrak {S}}(V)$ and ${{\mathbb {C}}}\llbracket Z_1,\ldots ,Z_m \rrbracket$ can be endowed with natural Fréchet algebra structures. Indeed the first is a closed subalgebra of $C^\infty (V)$ whereas for the second we consider its usual algebra structure endowed with its Fréchet topology defined by the seminorms $\sum _{\alpha }a_\alpha Z^\alpha \mapsto |a_\beta |, \quad \beta \in {{\mathbb {Z}}}^m_+$. Furthermore ${\mathfrak {b}}_V$ is a homomorphism of Fréchet algebras, a consequence of the Leibniz rule, and has dense image since it contains ${{\mathbb {C}}}[Z_1,\ldots ,Z_m]$.

Let ${\mathfrak {S}}_\infty (V,0)$ denote the ideal of ${\mathfrak {S}}(V)$ formed by all $v\in {\mathfrak {S}}(V)$ which vanish to infinite order at the origin. Thus $\ker {\mathfrak {b}}_V= {\mathfrak {S}}_\infty (V;0)$ and hence if ${\mathfrak {b}}_V$ is surjective we obtain an isomorphism of Fréchet algebras

$$\begin{aligned} {\mathfrak {S}}(V)/{\mathfrak {S}}_\infty (V,0) \simeq {{\mathbb {C}}}\llbracket Z_1,\ldots ,Z_m \rrbracket . \end{aligned}$$

Notice that ${\mathfrak {b}}_V$ can never be an isomorphism for ${{\mathbb {C}}}\llbracket Z_1,\ldots ,Z_m \rrbracket$ is a local algebra whereas the spectrum of ${\mathfrak {S}}(V)$ is not an unitary set: if $(x_0,t_0)\in V$ is such that $Z_j(x_0,t_0)\ne 0$ for some $j\in \{1,\ldots ,m\}$ then the Dirac measure at $(x_0,t_0)$ defines continuous homomorphism of ${\mathfrak {S}}(V)$ which is different from the Dirac measure at the origin (cf. Theorem 3.1 in [3]). In general the spectrum of the Fréchet algebra ${\mathfrak {S}}(V)/{\mathfrak {S}}_\infty (V,0)$ equals the set of all nonzero continuous homomorphisms ${\mathfrak {S}}(V)\rightarrow {{\mathbb {C}}}$ that vanish on ${\mathfrak {S}}_\infty (V;0)$ [7, pp. 81–82]. Hence when ${\mathfrak {b}}_V$ is surjective the only homomorphism ${\mathfrak {S}}(V)\rightarrow {{\mathbb {C}}}$ that vanishes on ${\mathfrak {S}}_\infty (V;0)$ is the Dirac measure at the origin.

**B.** Both ${\mathfrak {S}}(V)$ and ${{\mathbb {C}}}\llbracket Z_1,\ldots ,Z_m \rrbracket$ are also Fréchet-Montel spaces. Indeed the former is a closed subspace of the Fréchet-Montel space $C^\infty (V)$ and the latter is isomorphic to a countable product of copies of the complex field, which is easily seen to be Fréchet-Montel (Tychonoff theorem). We will make use the following result:

### Proposition 3.1

Let *E*, *F* be Fréchet-Montel spaces and let $A:E\rightarrow F$ be a continuous linear map with *A*(*E*) dense in *F*. The following properties are equivalent:

1. $$A(E)=F$$;
2. $\ ^tA(F')$ is strongly closed;
3. $$\forall {{\mathcal {B}}} \subset F'$$, $$\ ^tA({{\mathcal {B}}})\subset E'$$ strongly bounded $$\Rightarrow $$$${{\mathcal {B}}}$$ strongly bounded;
4. $$\forall \{y'_j\}\subset F'$$, $$\{\ ^tA(y'_j)\}$$ strongly bounded $$\Rightarrow $$$$\{y'_j\}$$ is strongly bounded;
5. $\ ^tA(F')$ is strongly sequentially closed in $E'$.

### Proof

The equivalence of (1) and (2) follows from [9], p. 22. The equivalence of (1) and (5) follows from [9], p. 18. Now, since $\ ^tA$ is injective, (2) implies that $(\ ^tA)^{-1}:\ ^tA(F')\rightarrow F'$ is continuous with respect to the strong topologies and then it maps strongly bounded sets into strongly bounded sets, which gives (3). It is clear that (3) implies (4). Assume now that (4) holds and let $\{\ ^tA(y'_j)\}\subset \ ^tA(F')$, $\ ^tA(y'_j)\rightarrow x'$ strongly in $E'$. By (4) $\{y'_j\}$ is strongly bounded in $E'$. Since $E'$, endowed with the strong topology, is also a Montel space, it follows that $\overline{\{y'_j\}}$ is compact, which in particular implies that $\overline{\{\ ^tA(y'_j)\}}=\ ^tA\overline{\{y'_j\}}$. Then $x'\in \ ^tA(E')$, which proves (5).

We apply Proposition 3.1 with $A={\mathfrak {b}}_V$, $E={\mathfrak {S}}(V)$, $F={{\mathbb {C}}}\llbracket Z_1,\ldots ,Z_m \rrbracket$. The dual of ${{\mathbb {C}}}\llbracket Z_1,\ldots ,Z_m \rrbracket$ is the space ${{\mathbb {C}}}[Z_,\ldots ,Z_m]$ under the duality

$$\begin{aligned} {{\mathbb {C}}}\llbracket Z_1,\ldots ,Z_m \rrbracket \times {{\mathbb {C}}}[Z_1,\ldots ,Z_m]\rightarrow {{\mathbb {C}}},\quad \left( \sum _{\alpha }a_\alpha Z^\alpha , \sum _{\mathrm{finite}} b_\alpha Z^\alpha \right) \mapsto \sum a_\alpha \,b_\alpha . \end{aligned}$$

Hence the transpose of ${\mathfrak {b}}_V$ is the map ${{\mathbb {C}}}[Z_,\ldots ,Z_m]\ni P\mapsto \lambda _P\in {\mathfrak {S}}(V)'$,

$$\begin{aligned} \lambda _P(f) = ({{\tilde{P}}}(M)f)(0), \quad f\in {\mathfrak {S}}(V), \end{aligned}$$

where ${\tilde{P}}$ is the polynomial obtained from *P* after dividing its coefficient $b_\alpha$ by $\alpha !$. Thus the Borel map ${\mathfrak {b}}_V$ is surjective if and only if given any sequence of polynomials $P_j\in {{\mathbb {C}}}[Z_1,\ldots ,Z_m]$ with $\lambda _{P_j}$ bounded in ${\mathfrak {S}}(V)'$ then $P_j$ is bounded in ${{\mathbb {C}}}[Z_1,\ldots , Z_m]$.

Now a sequence $P_j$ is bounded in ${{\mathbb {C}}}[Z_1,\ldots ,Z_m]$ if and only if there is *k* such that $\hbox {degree}(P_j)\le k$ for every *j* and the sequences of the corrresponding coefficients are bounded in ${{\mathbb {C}}}$. On the other hand the sequence $\lambda _{P_j}$ is bounded in ${\mathfrak {S}}(V)'$ if and only if it is equicontinuous, that is

3.1 $$\begin{aligned} \begin{aligned} \textit{There are an open set } 0\in W\subset \subset V, \ell \in {\mathbb {Z}}_+ \textit{ and } C>0 \textit{ such that} \\ |({\tilde{P}}_j(M)f)(0)|\le C\Vert f\Vert _{C^\ell ({\bar{W}})}, \quad f\in {\mathfrak {S}}(V),\,\,j\ge 1. \end{aligned} \end{aligned}$$

Notice that applying (3.1) to the monomials $f=Z^\beta$ implies that the sequence of corresponding coefficients of $P_j$ is bounded in ${{\mathbb {C}}}$. We summarize:

### Proposition 3.2

${\mathfrak {b}}_V$ is surjective if and only if the following holds: given any sequence of polynomials $P_j\in {{\mathbb {C}}}[Z_1,\ldots ,Z_m]$ satisfying (3.1) then $\sup \{\mathrm {degree}(P_j)\}<\infty$.

## The partial Borel maps

**A.** We keep the notation established in the previous section and start with a digression regarding the theory of tensor products in the category of Fréchet spaces.

Let $1\le p<m$ and consider the natural inclusions

$$\begin{aligned} {{\mathbb {C}}}\llbracket Z_1,\ldots ,Z_p \rrbracket \hookrightarrow {{\mathbb {C}}}\llbracket Z_1,\ldots ,Z_m \rrbracket , \quad {{\mathbb {C}}}\llbracket Z_{p+1},\ldots ,Z_m \rrbracket \hookrightarrow {{\mathbb {C}}}\llbracket Z_1,\ldots ,Z_m \rrbracket . \end{aligned}$$

Then ${{\mathbb {C}}}\llbracket Z_1,\ldots ,Z_p \rrbracket \otimes {{\mathbb {C}}}\llbracket Z_{p+1},\ldots ,Z_m \rrbracket$ can be identified to the (dense) subspace of ${{\mathbb {C}}}\llbracket Z_1,\ldots ,Z_m \rrbracket$ formed by all power series of the form

$$\begin{aligned} \sum _{j=1}^M S_{1,j}(Z_1,\ldots ,Z_p)S_{2,j}(Z_{p+1},\ldots ,Z_{m}). \end{aligned}$$

Recall that ${{\mathbb {C}}}\llbracket Z_1,\ldots ,Z_p \rrbracket {\widehat{\otimes }}_\pi {{\mathbb {C}}}\llbracket Z_{p+1},\ldots ,Z_m \rrbracket$ is the completion of this space endowed with the strongest locally convex topology which makes the natural map

$$\begin{aligned} B:{{\mathbb {C}}}\llbracket Z_1,\ldots ,Z_p \rrbracket \times {{\mathbb {C}}}\llbracket Z_{p+1},\ldots ,Z_m \rrbracket \rightarrow {{\mathbb {C}}}\llbracket Z_1,\ldots ,Z_p \rrbracket \otimes {{\mathbb {C}}}\llbracket Z_{p+1},\ldots ,Z_m \rrbracket \end{aligned}$$

continuous. On the other hand since the space of formal power series is nuclear [12], p. 526, Corollary 1, it follows from [12], p. 511, Theorem 50.1 that the canonical map of

$$\begin{aligned} {{\mathbb {C}}}\llbracket Z_1,\ldots ,Z_p \rrbracket {\widehat{\otimes }}_\pi {{\mathbb {C}}}\llbracket Z_{p+1},\ldots ,Z_m \rrbracket \longrightarrow {{\mathbb {C}}}\llbracket Z_1,\ldots ,Z_p \rrbracket {\widehat{\otimes }}_\varepsilon {{\mathbb {C}}}\llbracket Z_{p+1},\ldots ,Z_m \rrbracket \end{aligned}$$

is an isomorphism (cf. the definition of the $\varepsilon$ topology in [12], page 434). In other words both $\pi$ and $\varepsilon$ topologies coincide. If we apply the same reasoning as in the proof of [12], p. 531, Theorem 51.6, it follows that ${{\mathbb {C}}}\llbracket Z_1,\ldots ,Z_p \rrbracket {\widehat{\otimes }} {{\mathbb {C}}}\llbracket Z_{p+1},\ldots ,Z_m \rrbracket \cong {{\mathbb {C}}}\llbracket Z_1,\ldots ,Z_m \rrbracket$.

By a property of the $\pi$-topology [14], Theorem 6.4, p. 63, it then follows that every element $S\in {{\mathbb {C}}}\llbracket Z_1,\ldots ,Z_m \rrbracket$ can be represented in the form

4.1 $$\begin{aligned} S=\sum _{j=1}^\infty S_{1,j}(Z_1,\ldots ,Z_p)S_{2,j}(Z_{p+1},\ldots ,Z_{m}), \end{aligned}$$

where

$$\begin{aligned} \sum _{j=1}^\infty q_k(S_{1,j})q_k(S_{2,j}) < 1 \end{aligned}$$

and $q_1<q_2<\ldots$ is a sequence of continuous seminorms that define the Fréchet topology in ${{\mathbb {C}}}\llbracket Z_1,\ldots ,Z_m \rrbracket$.

**B.** Denote by ${\mathfrak {S}}^{(1)}_0$ (resp. ${\mathfrak {S}}_0^{(2)}$) the space of all $u\in {\mathfrak {S}}_0$ such that ${\mathrm {M}}^\alpha u(0)=0$ if $\alpha \not \subset \{1,\ldots ,p\}$ (resp. $\alpha \not \subset \{p+1,\ldots ,n\}$). We then obtain homomorphisms induced by ${\mathfrak {b}}$:

$$\begin{aligned} {\mathfrak {b}}_1 :{\mathfrak {S}}^{(1)}_0\rightarrow {{\mathbb {C}}}\llbracket Z_1,\ldots ,Z_p \rrbracket , \,\,{\mathfrak {b}}_2 :{\mathfrak {S}}_0^{(2)} \rightarrow {{\mathbb {C}}}\llbracket Z_{p+1},\ldots ,Z_m \rrbracket . \end{aligned}$$

We shall refer to the maps ${\mathfrak {b}}_\ell$ as the * partial Borel maps* for ${\mathcal {V}}$ at the origin with the respect to the decomposition $\{1,\ldots ,m\}=\{1,\ldots ,p\}\cup \{p+1,\ldots ,m\}$.

### Theorem 4.1

The Borel map ${\mathfrak {b}}$ is surjective if and only if each ${\mathfrak {b}}_\ell$ is surjective, $\ell =1,2$.

### Proof

If ${\mathfrak {b}}$ is surjective and if $S\in {{\mathbb {C}}}\llbracket Z_1,\ldots ,Z_p \rrbracket \subset {{\mathbb {C}}}\llbracket Z_1,\ldots ,Z_m \rrbracket$ then there is $u\in {\mathfrak {S}}_0$ such that ${\mathfrak {b}}(u)=S$. But a fortiori $u\in {\mathfrak {S}}_0^{(1)}$ by the definition of ${\mathfrak {b}}$ and thus ${\mathfrak {b}}_1(u)={\mathfrak {b}}(u)=S$, which shows that ${\mathfrak {b}}_1$ is surjective. An analogous argument shows the surjectivity of ${\mathfrak {b}}_2$.

We show the converse. Firstly we remark that if *V* is an open neighborhood of the origin and if we denote by ${\mathfrak {S}}^{(\ell )}(V)$, $j=1,2$, the space of all $u\in {\mathfrak {S}}(V)$ such that the germ of *u* at the origin belongs the ${\mathfrak {S}}^{(\ell )}_0$ then each ${\mathfrak {S}}^{(\ell )}(V)$ is a closed subspace of ${\mathfrak {S}}(V)$ and hence also a Fréchet space.

By a Baire category argument (cf. Lemma 3.2 in [3]) there is an open neighborhood *V* of the origin such that both induced maps

$$\begin{aligned} {\mathfrak {b}}_{1,V}:{\mathfrak {S}}^{(1)}(V) \rightarrow {{\mathbb {C}}}\llbracket Z_1,\ldots ,Z_p \rrbracket , \,\,\, {\mathfrak {b}}_{2,V}:{\mathfrak {S}}^{(2)}(V) \rightarrow {{\mathbb {C}}}\llbracket Z_{p+1},\ldots ,Z_m \rrbracket \end{aligned}$$

are surjections between Fréchet spaces. From [14], Theorem 6.6, p. 65, it follows that

$$\begin{aligned} {\mathfrak {b}}_{1,V}{\widehat{\otimes }} {\mathfrak {b}}_{2,V}: {\mathfrak {S}}^{(1)}(V) \otimes _\pi {\mathfrak {S}}^{(2)}(V)\longrightarrow {{\mathbb {C}}}\llbracket Z_1,\ldots ,Z_p \rrbracket {\widehat{\otimes }}_\pi {{\mathbb {C}}}\llbracket Z_{p+1},\ldots ,Z_m \rrbracket \end{aligned}$$

is a surjection between Fréchet spaces.

Thus by [14], Theorem 6.5, p. 63, given *S* as in (4.1) there are $u_j\in {\mathfrak {S}}^{(1)}(V)$, $v_j\in {\mathfrak {S}}^{(2)}(V)$ such that $\sum _{j=1}^\infty u_j(x,t)v_j(y,t)$ converges in $C^\infty (V\times V)$ and such that

$$\begin{aligned} S = \sum _{j=1}^\infty {\mathfrak {b}}_1(u_j){\mathfrak {b}}_2(v_j). \end{aligned}$$

Now since each ${\mathfrak {b}}_j$ is defined as the restriction of ${\mathfrak {b}}$ we can further write

$$\begin{aligned} S = \sum _{j=1}^\infty {\mathfrak {b}}(u_j){\mathfrak {b}}(v_j)= \sum _{j=1}^\infty {\mathfrak {b}}(u_jv_j), \end{aligned}$$

since ${\mathfrak {b}}$ is an algebra homomorphism. But then if we set $u(x,t){\doteq } \sum _{j=1}^\infty u_j(x,t)v_j(x,t)$ then $u\in {\mathfrak {S}}(V)$ and ${\mathfrak {b}}(u)=S$, which completes the proof.

Still keeping the notation previously established we consider the locally integrable structure ${\mathcal {V}}_1$ over *U* defined as ${\mathcal {V}}_1^\perp = \hbox {span}\{\hbox {d}Z_1,\ldots ,{\mathrm {Z}}_p\}$. Notice that a *u* is a solution for ${\mathcal {V}}_1$ if and only if

$$\begin{aligned} {\mathrm {L}}_j u=0, \,\, {\mathrm {M}}_k u=0,\quad j=1,\ldots ,n, \,\, k=p+1,\ldots , n. \end{aligned}$$

In particular ${\mathrm {M}}^\alpha u=0$ in a full neighborhood of the origin if $\alpha \not \subset \{1,\ldots ,p\}$ and consequently the following statement is immediate:

### Proposition 4.1

If the Borel map for ${\mathcal {V}}_1$ at the origin is surjective then the same is true for the partial Borel map ${\mathfrak {b}}_1$.

## Partial hypocomplexity

In this section we continue to write $Z(x,t)=(Z_1(x,t),\ldots ,Z_m(x,t))\in {{\mathbb {C}}}^m$ and remark that for a fixed structure ${\mathcal {V}}$ all concepts below are independent of a particular choice of such map.

**A.** In the first paragraph of this section we recall the concept of hypocomplexity and some results presented in [12]. Denote by ${\mathcal {O}}^{(m)}$ the sheaf of germs of holomorphic functions at the origin in ${{\mathbb {C}}}^m$. We say that ${\mathcal {V}}$ is * hypocomplex at the origin* if every germ of (weak) solution *u* for ${\mathcal {V}}$ at the origin can be written as $u=H\circ Z$ for some $H\in {\mathcal {O}}^{(m)}$. In this case given any solution *u* for ${\mathcal {V}}$ defined near the origin we have, for some constant $C>0$, $|{\mathrm {M}}^\alpha u(0)|\le C^{|\alpha |+1}\alpha !, \,\, \alpha \in {{\mathbb {Z}}}^m_+,$ and consequently hypocomplexity at the origin implies the non surjectivity of the Borel map.

The following theorem gives a complete characterization of hypocomplexity in terms of the compact neighborhoods of the origin in *U*. If we recall that for a compact set $P\subset {{\mathbb {C}}}^m$ its rational hull can be characterized as the set all $z\in {{\mathbb {C}}}^m$ having the following property: * every algebraic hypersurface through z intersects P*, we can state Theorem III.5.1 in [12] in the following form:

### Theorem 5.1

The following properties are equivalent:

1. ${\mathcal {V}}$ is hypocomplex at the origin;
2. For every compact neighborhood $K_0\subset \subset U$ of the origin in ${{\mathbb {R}}}^N$ the rational hull of $Z( K_0)$ is a neighborhood of the origin in ${{\mathbb {C}}}^m$;
3. For every compact neighborhood $K_0\subset \subset U$ of the origin in ${{\mathbb {R}}}^N$ the polynomial hull of $Z( K_0)$ is a neighborhood of the origin in ${{\mathbb {C}}}^m$.

As a consequence we obtain:

### Corollary 5.1

If ${\mathcal {V}}$ is hypocomplex at the origin then any non constant solution near the origin is open at the origin.

For a proof see ([12], Corollary III.5.2).

### Corollary 5.2

Assume $m=1$. Then ${\mathcal {V}}$ is hypocomplex at the origin if and only if *Z* is open at the origin.

### Proof

The rational hull of any compact set in ${{\mathbb {C}}}$ is the compact itself.

**B.** Recall that if ${\mathcal {V}}$ is a locally integrable structure over $\varOmega$ its characteristic set is the subset of ${\mathrm {T}}^*\varOmega$ defined by ${\mathrm {T}}^{\mathrm {o}}= {\mathcal {V}}^\perp \cap {\mathrm {T}}^*\varOmega$.

Taking into account Corollary 5.1, and for further reference, we conclude this section introducing a weakened version of hypocomplexity:

### Definition 5.1

We shall say that ${\mathcal {V}}$ is partially hypocomplex at the origin if there is a smooth solution *W* for ${\mathcal {V}}$ near the origin, with $\hbox {d}W|_0 \in {\mathrm {T}}^{\mathrm {o}}_0\setminus 0$, such that *W* is open at the origin.

### Remark 5.1

Write the coordinates in ${{\mathbb {C}}}^2$ as $z=x+iy$, $w=s+it$ and consider the hypersurface $\varSigma$ defined by $t=s|z|^2$. The CR structure ${\mathcal {V}}$ on $\varSigma$ is such that its orthogonal is spanned by the differentials of the functions $Z_1=x+iy$, $Z_2= s + is|z|^2$. The characteristic set at the origin is spanned by $\hbox {d}s|_0$ and the function $W=Z_2+iZ_1^2$ is a solution with $\hbox {d}W(0) = \hbox {d}s|_0$. Moreover introducing $s'=s-2xy$ as a new variable we have

$$\begin{aligned} W(x,y,s') = s' + i (x^2-y^2 + (s'+2xy)(x^2+y^2)) \end{aligned}$$

and then $({\mathsf {Im}} W)(x,y,0)$ changes sign at the origin in ${{\mathbb {R}}}^2$. Hence *W* is open at the origin and consequently this CR structure is partially hypocomplex (but not hypocomplex) at the origin. $\square$

## A necessary condition for the surjectivity of the Borel map

In the preceding section we have seen that when $\widehat{Z(K)}$ is a neighborhood of the origin ${{\mathbb {C}}}^m$ ($K\subset U$ a compact neighborhood of the origin) the Borel map is not surjective. We now prove a much stronger statement:

### Theorem 6.1

Suppose that for every $K\subset U$ compact neighborhood of the origin the polynomial hull $\widehat{Z(K)}$ of *Z*(*K*) in ${{\mathbb {C}}}^m$ contains a non constant complex curve through the origin. Then the Borel map for ${\mathcal {V}}$ at the origin is not surjective.

### Proof

Let *u* be a solution for ${\mathcal {V}}$ defined near the origin. There are a compact neighborhood *K* of the origin in ${{\mathbb {R}}}^N$ and a sequence of polynomials $P_\nu \in {{\mathbb {C}}}[z_1,\ldots ,z_m]$ such that $P_\nu \circ Z$ converges to *u* over *K* in the $C^\infty$ topology (the Baouendi–Treves approximation theorem). In particular $P_\nu$ converges uniformly over *Z*(*K*). Now by hypothesis there is a non constant complex curve $\tau \mapsto \gamma (\tau )\in \widehat{Z(K)}$, defined near the origin in the complex plane and such that $\gamma (0)=0$. Hence $P_\nu (\gamma (\tau ))$ converges uniformly to a holomorphic function $\alpha (\tau )$ in a neighborhood of the origin in ${{\mathbb {C}}}$. In particular

6.1 $$\begin{aligned} \frac{\hbox {d}^k}{\hbox {d}\tau ^k}P_\nu (\gamma (\tau ))|_{\tau = 0} \rightarrow \alpha ^{(k)}(0) \end{aligned}$$

for every *k*. On the other hand, the Faà di Bruno formula gives

$$\begin{aligned}&\frac{\hbox {d}^k}{\hbox {d}\tau ^k}P_\nu (\gamma (\tau ))|_{\tau = 0} = \sum _{1\le |\alpha |\le k} \varLambda _{\alpha ,k} (\partial _z^\alpha P_\nu )(0),\\&\varLambda _{\alpha ,k} \doteq \sum _{s=1}^k \sum _{p_s(\alpha ,k)} k! \prod _{j=1}^s \frac{ [\gamma ^{(\ell _j)}(0)]^{\alpha _j}}{\alpha _j!\ell _j!^{|\alpha _j|}} \end{aligned}$$

where $p_s(\alpha ,k)$ is the set of all $(\alpha _1,\ldots ,\alpha _s,\ell _1,\ldots ,\ell _s)\in ({{\mathbb {Z}}}_+^m)^s\times {{\mathbb {Z}}}_+^s$ satisfying $|\alpha _j|>0$, $\sum \alpha _j=\alpha$ and $\sum |\alpha _j|\ell _j=k$.

By hypothesis there is $r\ge 1$ such that

$$\begin{aligned} \gamma (\tau ) = \tau ^r \gamma _\bullet (\tau )/r!,\quad \zeta \doteq \gamma _\bullet (0)\ne 0. \end{aligned}$$

Thus $\gamma ^{(j)}(0)=0$ if $j\le r-1$ and $\gamma ^{(r)}(0)=\zeta \ne 0$.

We assume $k=rq$, where $q=1,2,\ldots$ and consider two cases:

- Case 1: $|\alpha |>q$. If $(\alpha _1,\ldots ,\alpha _s,\ell _1,\ldots ,\ell _s)\in p_s(\alpha ,rq)$ we have $\sum _{j}|\alpha _j|\ell _j<|\alpha |r$. Hence $\ell _j<r$ for some *j* and thus $\varLambda _{\alpha ,rq}=0$.   $\Box$
- Case 2.: $|\alpha |=q$. f $(\alpha _1,\ldots ,\alpha _s,\ell _1,\ldots ,\ell _s)\in p_s(\alpha ,rq)$ we have $\sum _{j}|\alpha _j|\ell _j=|\alpha |r$. Hence if $\ell _j\ge r$ for every *j* we necessarily must have $\ell _j=r$ for every *j*$\square$

Summing up when $k=rq$ we conclude that $\varLambda _{\alpha ,rq}=0$ if $|\alpha |>q$ and

$$\begin{aligned} \varLambda _{\alpha ,rq} = (rq)!\sum _{s=1}^{rq} \sum _{\begin{array}{c} \sum _{j=1}^s \alpha _j =\alpha \\ {\alpha _j\ne 0} \end{array}} \prod _{j=1}^s \frac{ [\gamma ^{(r)}(0)]^{\alpha _j}}{\alpha _j! r!^{|\alpha _j|}} \doteq A_{\alpha ,q} \zeta ^{\alpha } \quad \hbox { if } \,\,|\alpha |=q, \end{aligned}$$

where $A_{\alpha ,q}$ is a positive constant. Thus

$$\begin{aligned} \frac{\hbox {d}^{rq}}{\hbox {d}\tau ^{rq}}P_\nu (\gamma (\tau ))|_{\tau = 0} = \sum _{|\alpha |=q} A_{\alpha , q} \zeta ^\alpha (\partial _z^\alpha P_\nu )(0) + Q_{q}(\partial _z)P_\nu (0) \end{aligned}$$

where $Q_{q}(X)=\sum _{|\beta |\le q-1}Q_{q,\beta }X^\beta /\beta !\in {{\mathbb {C}}}[X_1,\ldots ,X_m]$ has degree $\le q-1$.

Now since

$$\begin{aligned} (\partial ^\alpha P_\nu /\partial z^\alpha )(0) = {\mathrm {M}}^\alpha \left\{ P_\nu \circ Z\right\} |_{(x,t)=(0,0)} \end{aligned}$$

from (6.1) we obtain

$$\begin{aligned} \alpha ^{(rq)}(0) = \sum _{|\alpha |=q} A_{\alpha , q} \zeta ^\alpha ({\mathrm {M}}^\alpha u)(0) + Q_{rq}({\mathrm {M}})u(0) \end{aligned}$$

and consequently for some constant $C>0$ we have

$$\begin{aligned} \left| \sum _{|\alpha |=q} A_{\alpha , q} \zeta ^\alpha ({\mathrm {M}}^\alpha u)(0) + Q_{q}({\mathrm {M}})u(0)\right| \le C^{q+1}(rq)!. \end{aligned}$$

In particular, if $\sum _{\beta } a_\beta Z(x,t)^\beta /\beta !\in {{\mathbb {C}}}\llbracket Z_1,\ldots ,Z_m \rrbracket$ belongs to the image of the Borel map for ${\mathcal {V}}$ at the origin then

$$\begin{aligned} \left| \sum _{|\alpha |=q} A_{\alpha ,q}{a_\alpha } \zeta ^\alpha + \sum _{|\beta |\le q-1} Q_{q,\beta }a_{\beta } \right| \le C^{q+1}(rq)!, \quad k\ge 0, \end{aligned}$$

for some $C>0$. Since it is easy to construct indutively a sequence $(a_\beta )$ for which this property is not satisfied for any $C>0$ our proof is complete.

### Remark 6.1

Our argument in the proof of Theorem 6.1 can be enlightened by the following discussion. Given a formal curve $\gamma (t) \in {{\mathbb {C}}}\llbracket t \rrbracket ^m$, with $\gamma (0)=0$, the map

$$\begin{aligned} \gamma ^* : {{\mathbb {C}}}\llbracket z_1,\ldots ,z_m \rrbracket \longrightarrow {{\mathbb {C}}}\llbracket t \rrbracket , \quad u\mapsto u\circ \gamma , \end{aligned}$$

is onto if $\gamma ' (0) \ne 0$. More generally, if $\gamma (t) = t^{d}\delta (t)$ with $\delta (0)\ne 0$, and if we consider the projection map

$$\begin{aligned} \pi :{{\mathbb {C}}}\llbracket t \rrbracket \longrightarrow {{\mathbb {C}}}\llbracket t^d \rrbracket , \quad \pi \left( \sum _j \alpha _j t^j\right) = \sum _k \alpha _{dk } t^{dk}, \end{aligned}$$

then

$$\begin{aligned} \pi \circ \gamma ^* :{{\mathbb {C}}}\llbracket z_1,\ldots ,z_m \rrbracket \longrightarrow {{\mathbb {C}}}\llbracket t^d \rrbracket \end{aligned}$$

is onto, since by the Faà di Bruno formula, for each *k* there exists a polynomial $$p_k $$ such that the coefficient of $$t^{dk}$$ in $$(\pi \circ \gamma ^*) ( \sum _{\alpha } a_\alpha Z^\alpha )$$ can be written as

$$\begin{aligned} \sum _{|\alpha |=k} a_\alpha \delta (0)^\alpha + p_k (a_\beta , \delta _j :|\beta | < k, j\le dk). \end{aligned}$$

Theorem 6.1 shows that if $\gamma$ happens to be an analytic curve contained in $\widehat{Z(K)}$, then by the Baouendi–Treves approximation theorem,

$$\begin{aligned} (\pi \circ \gamma ^*) \left( {\mathfrak {b}}({\mathfrak {S}}_0)\right) \subset {{\mathbb {C}}}\{t^d\} \end{aligned}$$

and hence the Borel property must fail. $\square$

### Remark 6.2

For the CR structure defined in Remark 5.1 the Borel map at the origin is not surjective since the complex curve $w=0$ is contained $\varSigma$.

### Remark 6.3

Write the coordinates in ${{\mathbb {C}}}^3$ as $z_j=x_j+iy_j$, $j=1,2$, and $w=s+it$ and consider the hypersurface $\varSigma$ defined by

$$\begin{aligned} t = \left| z_1^2 -z_2^3 \right| ^2. \end{aligned}$$

Let ${\mathcal {V}}$ be the CR structure on $\varSigma$ induced by the complex structure in ${{\mathbb {C}}}^3$. Since $\varSigma$ contains the germ of the curve $\zeta \mapsto (\zeta ^3,\zeta ^2,0)$ it follows from Theorem 6.1 that the Borel map for ${\mathcal {V}}$ at the origin is not surjective. We do conjecture that the polynomial hull of a compact neighbourhood of 0 in *M* also does not contain any regular curve. For such a compact neighborhood of the origin $K\subset \varSigma$ in $\varSigma$ it can be shown (see [5]) that the the analogous question for the *holomorphic hull* of *K* has an affirmative answer, that is, the holomorphic hull of *K* does not contain any germ of a * regular* curve curve through the origin.

## Sufficient conditions for the surjectivity of the Borel map

In this section we recall two conditions which imply the surjectivity of the Borel map.

**A.** Here we assume that ${\mathcal {V}}$ defines on $\varOmega$ a CR structure of the hypersurface type. Hence we have ${\mathcal {V}}\cap {\overline{{\mathcal {V}}}}=0$ and ${\mathrm {T}}^{\mathrm {o}}$ is a real line subbundle of ${\mathrm {T}}^*\varOmega$. Let $p\in \varOmega$, let $V\subset \varOmega$ be an open neighborhood of *p* and let $\psi \in {\mathfrak {S}}(V)$. We say that $\psi$ is a * peak function at p* if $\psi (p)=0$, $\psi (q)\ne 0$ for $q\ne p$ and $\arg \psi \ne -\pi$ in $V{\setminus }\{p\}$. Furthermore, we say that a peak function if of * finite type* if $|\psi (q)|\ge Cd(q,p)^\alpha$ for positive constants *C* and $\alpha$.

The following theorem is the main result in [6] :

### Theorem 7.1

If ${\mathcal {V}}$ is a CR structure of the hypersurface type in $\varOmega$ which admits a peak function of finite type at $p\in \varOmega$ then the Borel map for ${\mathcal {V}}$ at *p* is surjective.

We shall present a sketch of a proof of this result based on the characterization given in Proposition 3.2. For this we shall show that given any sequence of polynomials $P_j\in {{\mathbb {C}}}[Z_1,\ldots ,Z_m]$ such that $\hbox {degree}(P_j)\rightarrow \infty$, given $0\in W\subset U$ a neighborhood of the origin and $\ell \in {{\mathbb {Z}}}_+$ there is a sequence $f_j \in {\mathfrak {S}}(W)$ such that

$$\begin{aligned} |({\tilde{P}}_j({\mathrm {M}})f_j)(0)|=1, \quad \Vert f_j\Vert _{C^\ell ({\bar{W}})} \rightarrow 0. \end{aligned}$$

We can assume that $\varOmega$ is a hypersurface embedded in ${{\mathbb {C}}}^m$ and that $Z_j=z_j|_\varOmega$, $j=1,\ldots ,m$, where $(z_1,\ldots ,z_m)$ are the holomorphic coordinates in ${{\mathbb {C}}}^m$. We can also assume that the peak function $\psi$ is defined in in *V*.

For any j let $b_{\alpha _j} z^{\alpha _j}$ be a non-vanishing monomial of $P_j$ of maximal degree, and define $C_{\alpha _j}=1/b_{\alpha _j}$. For any multiindex $\beta \in {{\mathbb {Z}}}_+^m$ we put $d_\beta =2^{-|\beta |}$. We define a sequence $f_j\in {\mathfrak {S}}(V)$ by $f_j=C_{\alpha _j}Z^{\alpha _j}\varphi _{\alpha _j}$, where $\varphi _{\alpha _j}\in {\mathfrak {S}}(V)$ is the function constructed in [6], Lemma 4.2. We have that $\varphi _{\alpha _j}(0)=1$ for all $j\in {\mathbb {N}}$, and all its derivative vanish at 0 (see [6] Corollary 4.3).

By [6] Lemma 5.1 and more in particular from equation (5.4) in [6], we have the following: for fixed $\beta \in {{\mathbb {Z}}}_+^m$ there exists $j_0(\beta )\in {{\mathbb {Z}}}_+$ such that $|{\mathrm {M}}^\beta f_j(q)|\le A|\alpha _j|^{|J|}d_{\alpha _j}$ for all $q\in V$ and $j\ge j_0(\beta )$ (here we are using the fact that $|\alpha _j|\rightarrow \infty$ as $j\rightarrow \infty$), where the constant *A* depends on $|\beta |$ but not on $\alpha _j$. Using these inequalities for all $\beta \in {{\mathbb {Z}}}_+^m$ with $|\beta |\le \ell$, it follows that there exist $j_1(\ell )\in {\mathbb {Z}}_+$ and $A_1=A_1(\ell )>0$ such that $\Vert f_j\Vert _{C^\ell (V)}\le A_1|\alpha _j|^\ell d_{\alpha _j}$. Thus $\Vert f_j\Vert _{C^\ell ({\bar{W}})}\rightarrow 0$ as $j\rightarrow \infty$ for any neighborhood $W\subset \subset V$ of the origin.

On the other hand, let us consider $({\widetilde{P}}_j({\mathrm {M}})f_j)(0)$. Since $({\mathrm {M}}_k\varphi _{\alpha _j})(0)=0$ for all *k*, it follows that $({\widetilde{P}}_j({\mathrm {M}})f_j)(0)=C_{\alpha _j}({\widetilde{P}}_j({\mathrm {M}})Z^{\alpha _j})(0)\varphi _{\alpha _j}(0)=C_{\alpha _j}({\widetilde{P}}_j({\mathrm {M}})Z^{\alpha _j})(0)$. Using that ${\mathrm {M}}_kZ_{k'}=\delta _{kk}$ it is clear that ${\mathrm {M}}^\beta Z^{\alpha _j}(0)=0$ for all $\beta \ne \alpha _j$, hence $C_{\alpha _j}({\widetilde{P}}_j({\mathrm {M}})Z^{\alpha _j})(0)=C_{\alpha _j}b_{\alpha _j}({\mathrm {M}}^{\alpha _j}Z^{\alpha _j})(0)/\alpha _j!=C_{\alpha _j}b_{\alpha _j}=1$, which completes the proof. $\square$

**B.** Next we introduce a very similar condition stated in [3] which now applies to an arbitrary locally integrable structure ${\mathcal {V}}$. We say that ${\mathcal {V}}$ satisfies condition ($\mathfrak {B}$) at $p\in \varOmega$ if there is a smooth solution *W* for ${\mathcal {V}}$ near *p* such that the following conditions holds:

1. $$W(p)=0$$, $$\hbox {d}W(p)\in {\mathrm {T}}^{\mathrm {o}}_p{\setminus } 0$$ and $$\hbox {arg}\, W\ne -\pi /2$$ near *p*;
2. There are smooth solutions $W_1,\ldots ,W_{m-1}$ defined in a neighborhood of *p*, $W_j(p)=0$, such that $\hbox {d}W_1,\ldots ,W_{m-1},\hbox {d}W$ are linearly independent and positive constants $\mu$ and *C* such that $(|W_1|+\cdots +|W_{m-1}|)^\mu \le C|W|$ near *p*.

The main result in [3] is the following:

### Theorem 7.2

Property ($\mathfrak {B}$) at *p* implies the surjectivity of the Borel map for ${\mathcal {V}}$ at *p*.

It is an easy corollary of Theorem 7.2 the fact that when ${\mathcal {V}}$ has rank $N-1$, that is when ${\mathcal {V}}^\perp$ is locally spanned by the differential of a single function, * the surjectivity of the Borel map at*$p\in \varOmega$* is equivalent to the fact that *${\mathcal {V}}$* is not hypocomplex at p* ([3], Corollary 6.2).

The conjunction of Theorem 4.1 and this result allows us to obtain the following statement:

### Theorem 7.3

Assume that the characteristic set for the locally integrable ${\mathcal {V}}$ over $\varOmega$ at $p\in \varOmega$ has maximum dimension ($=m$). If ${\mathcal {V}}$ is not partially hypocomplex at *p* then the Borel map for ${\mathcal {V}}$ at p is surjective.

### Proof

Since $\dim {\mathrm {T}}^{\mathrm {o}}_p=m$ by ( [4] Theorem I.10.1) we can find smooth solutions $Z_1,\ldots ,Z_m$ for ${\mathcal {V}}$ near *p* with $\hbox {d}Z_1,\ldots ,\hbox {d}Z_m$ linearly independent and $\hbox {d}Z_j(p)\in {\mathrm {T}}^{\mathrm {o}}_p$ for all $j=1,\ldots ,m$. By hypothesis none of the functions $Z_j$ is open at *p* and hence by Corollary 4.1 and the result just stated we conclude that the Borel maps for the structures ${\mathcal {V}}_j = \hbox {span}\, \{\hbox {d}Z_j\}^\perp$ are surjective at *p*. Hence Proposition 4.1 in conjunction with Theorem 4.1 gives the sought conclusion.

## A class of tubular structures

**A.** We recall (cf. [13], p. 308) that a locally integrable structure ${\mathcal {V}}$ over $\varOmega$ of rank *n* is * tubular* if given any point $p\in \varOmega$ there are an open neighborhood *U* of *p* and an abelian finite dimensional subalgebra $\mathfrak {g}$ of $C^\infty (U;{\mathrm {T}}\varOmega )$ such that $[{\mathfrak {g}},{\mathcal {V}}|_U]\subset {\mathcal {V}}|_U$, $\dim {\mathfrak {g}}_q = \dim {\mathfrak {g}}$ and ${{\mathbb {C}}}{\mathrm {T}}\varOmega _q= {\mathcal {V}}_q + {\mathfrak {g}}_q$ for all $q\in U$. Here

$$\begin{aligned} {\mathfrak {g}}_q=\{ {\mathrm {X}}_q: \, {\mathrm {X}}\in {\mathfrak {g}}\}\subset {\mathrm {T}}_q\varOmega ,\quad q\in U. \end{aligned}$$

It is proved in ([12], p. 308) that ${\mathcal {V}}$ is tubular if and only if given any point $p\in \varOmega$ there are, as in section 1(B), a coordinate system $(x_1,\ldots ,x_m,t_1,\ldots ,t_n)$ centered at *p* ($N=m+N$) and defined in an open neighborhood $U=B\times \varTheta$ of the origin in ${{\mathbb {R}}}^N$ and a smooth map $\varPhi =(\varPhi _1,\ldots ,\varPhi _m):\varTheta \rightarrow {{\mathbb {R}}}^m$ satisfying $\varPhi (0)=0$ such that ${\mathcal {V}}^\perp$ is spanned over *U* by the differential of the functions

$$\begin{aligned} Z_j(x,t) = x_j +\varPhi _j(t),\quad j=1,\ldots ,m. \end{aligned}$$

Observe that a set of *n* linearly independent vector fields which span ${\mathcal {V}}|_U$ is given by

$$\begin{aligned} {\mathrm {L}}_j = \frac{\partial }{\partial t_j} - i \sum _{k=1}^m \frac{\partial \varPhi _k}{\partial t_j}(t)\frac{\partial }{\partial x_k}\,, \quad j=1,\ldots ,n. \end{aligned}$$

Moreover since that in this particular case the vector fields ${\mathrm {M}}_k$ equal $\partial /\partial x_k$ the Borel map at the origin for ${\mathcal {V}}$ is given by

$$\begin{aligned} {\mathfrak {S}}_0 \ni u \mapsto {\mathfrak {b}}(u)=\sum _{\alpha \in {{\mathbb {Z}}}^m} \frac{(\partial _x^\alpha u)(0,0)}{\alpha !} \, Z(x,t)^\alpha . \end{aligned}$$

From now on we shall assume that

$$\begin{aligned} \varOmega \textit{ and }{\mathcal {V}}\textit{ are real-analytic}. \end{aligned}$$

In particular $\varPhi$ is a real-analytic map. The main reason for assuming such a hypothesis is that in this case hypocomplexity for ${\mathcal {V}}$ at the origin is perfectly determined: by a result due to Baouendi and Treves [2] this structure ${\mathcal {V}}$ is hypocomplex at the origin if and only if for every $\xi \in {{\mathbb {R}}}^m{\setminus }\{0\}$ the map $t\mapsto \varPhi (t)\cdot \xi$ is open at the origin.

**B.** Assume that $m=n+1$ and suppose that $\varPhi$ has the special form

$$\begin{aligned} \varPhi (t) = (t,\phi (t)) \end{aligned}$$

where $\phi :{{\mathbb {R}}}^n\rightarrow {{\mathbb {R}}}$ is real analytic, $\phi (0)=0$, $\hbox {d}\phi (0)=0$. Such structure ${\mathcal {V}}_\bullet$ is CR of the hypersurface type: indeed in this case it is the CR structure induced by the complex structure on ${{\mathbb {C}}}^{n+1}$, where the complex coordinates are written as $(z_1,\ldots ,z_{n+1})$, on the hypersurface defined by ${\mathsf {Im}}\,z_{m+1} = \phi ({\mathsf {Im}}\,z_1,\ldots ,{\mathsf {Im}}\,z_n)$. Notice that for this structure the CR vector fields read

$$\begin{aligned} {\mathrm {L}}_j = \frac{\partial }{\partial t_j} - i\frac{\partial }{\partial x_j} - i \frac{\partial \phi }{\partial t_j}(t)\frac{\partial }{\partial x_{m}}, \quad j=1,\ldots , m. \end{aligned}$$

Notice also that in this case

$$\begin{aligned} \xi \cdot \varPhi (t) = \sum _{j=1}^{m-1}\xi _jt_j + \xi _m\phi (t) \end{aligned}$$

which is open at the origin if $\xi _j\ne 0$ for some $j=1,\ldots ,m-1$. Hence ${\mathcal {V}}_\bullet$ is hypocomplex at the origin if and only if $\phi (t)$ is open at the origin.

We first study the case $n=1$, which is very simple. If ${\mathcal {V}}$ is not hypocomplex at the origin then either $\phi$ has a zero of even order at the origin or else $\phi$ vanishes identically. In the latter case we are in the Levi flat case in which case the Borel map at the origin is not surjective whereas that in the former case the argument in the proof of Theorem 8.1 below shows the existence of a peak function for ${\mathcal {V}}_\bullet$ at the origin and hence the surjectivity of the Borel map at the origin follows from Theorem 7.1.

In what follows we then assume that $n\ge 2$ and that $\phi$ does not vanish identically.

Our discussion of the surjectivity of the Borel map for this particular CR structure will be given in terms of the (germ of the) variety $V\doteq \phi ^{-1}\{0\}$. We start with the following result:

### Theorem 8.1

Let ${\mathcal {V}}_\bullet$, $\phi$ and *V* be as before.

1. If $\phi$ is open at the origin then the Borel map for ${\mathcal {V}}_\bullet$ is not surjective;
2. If $V=\{0\}$ then the Borel map for ${\mathcal {V}}_\bullet$ is surjective.

### Proof

We have already seen that if $\phi$ is open then ${\mathcal {V}}_\bullet$ is hypocomplex at the origin and hence (1) follows.

For (2) we can assume without loss of generality that $\phi >0$ outside the origin. Hence from the analiticity of $\phi$ we conclude that $\phi (t)\ge c|t|^{2q}$ if $|t|\le r$, where $c>0$, $r>0$ are small constants and $q\in {\mathbb {N}}$ . We set

$$\begin{aligned} \psi (x,t)= -i(x_m+i\phi (t)) + (x_m+i\phi (t))^2 +\kappa \sum _{j=1}^{m-1}(x_j+it_j)^{2q}, \end{aligned}$$

where $\kappa$ is positive small constant. It is clear that $\psi \in {\mathfrak {S}}(U)$. Furthermore if $r>0$ is chosen such that $\phi (t)\le 1/2$ if $|t|\le r$ then

$$\begin{aligned}&{\mathsf {Re}}\,\Psi (x,t) \ge x_m^2 + \phi (t)/2 +\kappa \sum _{j=1}^{m-1}{\mathsf {Re}}\,\{(x_j+it_j)^{2q}\} \ge x_m^2 + c|t|^{2q}/2\\&\quad +\kappa \sum _{j=1}^{m-1}{\mathsf {Re}}\,\{(x_j+it_j)^{2q}\} \end{aligned}$$

If we now use the elementary fact that for every $0<\varepsilon <1$ there is $C_\varepsilon >0$ (depending on *q*) such that

$$\begin{aligned} {\mathsf {Re}}\,(z^{2q}) \ge (1-\varepsilon )({\mathsf {Re}}\,z)^{2q} - C_\varepsilon ({\mathsf {Im}}\,z)^{2q}, \quad z\in {{\mathbb {C}}}, \end{aligned}$$

choosing $\varepsilon =1/2$ and $\kappa$ small enough gives

$$\begin{aligned} {\mathsf {Re}}\,\Psi (x,t) \ge x_m^2 + c|t|^{2q}/4 + \kappa (x_1^{2q}+\ldots + x_{m-1}^{2q})/2,\quad |t|\le r. \end{aligned}$$

Hence $\psi$ is a peak function of finite type for ${\mathcal {V}}_\bullet$ at the origin and then (2) follows from Theorem 7.1.

We have now to face the situation when $V\ne \{0\}$ and say $\phi \ge 0$. The former is equivalent to the existence of a (germ of a) non trivial real analytic curve $\gamma (s)$ through the origin in *t*-space over which $\phi$ vanishes identically. Notice that $\phi =0$ implies $\hbox {d}\phi =0$ (because $\phi \ge 0$) and hence also $\hbox {d}\phi$ vanishes on $\gamma$.

### Theorem 8.2

Let ${\mathcal {V}}_\bullet$, $\phi$ and *V* be as before. Assume that *V* contains the (germ of) a non trivial real analytic curve $\gamma (s)$ through the origin such that each of its components has a zero of odd order at the origin. Then the Borel map for ${\mathcal {V}}_\bullet$ at the origin is not surjective.

### Proof

Write $\gamma (s)=(\gamma _1(s),\ldots ,\gamma _n(s))$ and consider the tube structure ${\mathcal {V}}_\sharp$ on the (*x*, *s*)-space defined by the first integrals

$$\begin{aligned} Z^\sharp _j(x,t) = x_j + i \gamma _j(s),\quad j=1,\ldots ,n. \end{aligned}$$

This structure is defined by a single vector field, namely:

$$\begin{aligned} {\mathrm {L}}^\sharp = \frac{\partial }{\partial s} - \sum _{j=1}^n \gamma _j'(s) \frac{\partial }{\partial x_j}\, . \end{aligned}$$

The point for considering this new tube structure is the following key observation: * if u*(*x*, *t*) * is a smooth solution for *${\mathcal {V}}_\bullet$* near the origin then *$v(x,s)\doteq u(x_1,\ldots ,x_n,0,\gamma (s))$* is a smooth solution for *${\mathcal {V}}_\sharp$* near the origin, that is,*${\mathrm {L}}^\sharp v=0$. This follows from a simple computation.

Now if each $\gamma _j$ has an odd order zero at the origin then the map

$$\begin{aligned} s \mapsto \sum _{j=1}^n \gamma _j(s)\xi _j \end{aligned}$$

is open at the origin in ${{\mathbb {R}}}$ for any $\xi \in {{\mathbb {R}}}^n{\setminus } 0$, and consequently by the Baouendi–Treves [2] result alluded to above, it follows that ${\mathcal {V}}_\sharp$ is hypocomplex. Hence if *u* is any smooth solution for ${\mathcal {V}}_\bullet$ near the origin and if *v* is defined as above then we obtain the bounds

$$\begin{aligned} |\partial _{x}^\alpha u(0,0)|=|\partial _x^{\alpha } v(0,0)| \le C^{|\alpha |+1}\alpha ! ,\quad \alpha \in {{\mathbb {Z}}}^m_+, \end{aligned}$$

which imply that the Borel map for ${\mathcal {V}}_\bullet$ at the origin is not surjective.

**C.** In the rest of this section we shall focus on the case when $n=2$ and $\phi (t)=(t_1^p - t_2^q)^2$, $p,q\in {\mathbb {N}}$. Write $p/q=\alpha /\beta$, with $\alpha$ and $\beta$ without common factors. By Theorem 8.2 the Borel map for ${\mathcal {V}}_\bullet$ at the origin is not surjective if both $\alpha$ and $\beta$ are odd since $\phi$ vanishes on the curve $\gamma (s)=(s^\beta ,s^\alpha )$.

We shall now study some of the cases when $\alpha \ne \beta$ and either $\alpha$ or $\beta$ is even. We are able to settle the following situations:

### Theorem 8.3

Let $\phi$ be as before:

- if $q=2$ and *p* is odd then the Borel map is surjective;
- if $q=2$ and *p* is even then the Borel map is not surjective.

### Remark 8.1

In each one of the cases where the Borel map is not surjective, the necessary condition established in Theorem 6.1 is not satisfied (indeed, we prove the non-surjectivity precisely by applying Theorem 6.1).

We will first concentrate on the second statement.

**C1.** Given $k\in {\mathbb {N}}$, consider the following hypersurface of ${\mathbb {C}}^3$, which is equivalent to the ones introduced in subsection **B.** up to a complex linear change of coordinates:

$$\begin{aligned} \varSigma =\{x_3=(x_1^2-x_2^{2k})^2 \}. \end{aligned}$$

We also put $\varSigma _0=\varSigma \cap \{z_3=0\}$. Then $\varSigma _0$ can be seen as the union of the two hypersurfaces $S^+=\{x_1=x_2^k\}$ and $S^-=\{x_1=-x_2^k\}$, biholomorphic to each other. We want to show that the polynomial hull ${\widehat{\varSigma }}_0$ of $\varSigma _0$ in ${\mathbb {C}}^2$ (and thus the polynomial hull ${\widehat{\varSigma }}$ of $\varSigma$ in ${\mathbb {C}}^3$) contains a complex line passing through 0.

To this aim, we define $\varSigma _0'=\varSigma _0\cap \{x_2\ge 0\}$, $\varSigma _0''=\varSigma _0\cap \{x_2\le 0\}$: then we can write $\varSigma _0'=\{x_2=\root k \of {|x_1|}\}$ and $\varSigma _0''=\{x_2=-\root k \of {|x_1|}\}$. We claim that ${\widehat{\varSigma }}_0'$ contains (a neighborhood of 0 in) $\{(0,z_2)\in {\mathbb {C}}^2: x_2\ge 0\}$, and similarly ${\widehat{\varSigma }}_0''$ contains (a neighborhood of 0 in) $\{(0,z_2)\in {\mathbb {C}}^2: x_2\le 0\}$.

Choose then $c\in {\mathbb {C}}$, $c=a+ib$ with $0<a<(2k-1)/(2k)^{\frac{2k}{2k-1}}$ and define $f:{\mathbb {C}}\rightarrow {\mathbb {C}}^2$ as $f(\zeta )=(\zeta , c+\zeta ^2)$; furthermore define $\rho :{\mathbb {C}}^2\rightarrow {\mathbb {R}}$ as $\rho (z_1,z_2)=x_2-\root k \of {|x_1|}$. Writing $\zeta =u+iv$ we can express the composition $\rho \circ f:{\mathbb {C}}\rightarrow {\mathbb {R}}$ as $\rho \circ f(\zeta )= a - v^2 +u^2 - \root k \of {|u|}$.

Let now $\varphi :{\mathbb {R}}^+\rightarrow {\mathbb {R}}$ be defined as $\varphi (t)=\root k \of {t}-t^2$. A simple computation shows that $\varphi$ is strictly increasing on the interval $[0, 1/(2k)^{\frac{k}{2k-1}}]$ and $\varphi (1/(2k)^{\frac{k}{2k-1}})=(2k-1)/(2k)^{\frac{2k}{2k-1}}$. We can thus set $d'=\varphi ^{-1}(a)$ and, choosing $d'<d<1/(2k)^{\frac{k}{2k-1}}$, define the rectangle $R=\{u+iv:|u|< d, |v|< \sqrt{a+1}\}$.

With this choice of *R* we have that $\rho \circ f|_{\partial R}<0$. Indeed, whenever $|v|=\sqrt{a+1}$ we can write $\rho \circ f(\zeta )\le a - v^2=-1$, while for $|u|=d$ one has $\rho \circ f(\zeta )\le a - \varphi (|u|)\le a - \varphi (d)<0$ by the choice of *d*. On the other hand $\rho \circ f(0)=a>0$. It follows that the open set $U'=R\cap \{\rho \circ f>0\}$ is non-empty and relatively compact in *R*. The open set ${\mathbb {C}}{\setminus } {\overline{U}}'$ has a unique unbounded connected component *V*. Putting $U={\mathbb {C}}{\setminus } {\overline{V}}$, it follows that *U* is simply connected, $0\in U$ and $\partial U\subset \{\rho \circ f=0\}$.

We can thus consider $f:U\rightarrow {\mathbb {C}}^2$ as an analytic disc attached to $\varSigma _0'$ because $f(\partial U)\subset \varSigma _0'$. Since $f(0)=(0,c)$, it follows that $(0,c)\in {\widehat{\varSigma }}_0'$, which verifies the claim. By Theorem 6.1 we conclude that the Borel map is not surjective, which proves the second statement in Theorem 8.3.

**C2.** We are now going to treat the first claim in Theorem 8.3.

In order to do so we are going to study the properties of some particular domains of ${\mathbb {C}}^2$. Fix a small enough $\tau >0$ (to be specified later) and $k_0\in {\mathbb {N}}$. We define $\varOmega \subset {\mathbb {C}}^2(z_1,z_3)$ to be the set

$$\begin{aligned} \varOmega = \{(z_1,z_3)\in {\mathbb {C}}^2: x_3\ge 0, \ |z_3|<\tau , \ |z_1|<1+\root k_0 \of {x_3}\} \end{aligned}$$

and put $\varOmega _0=\varOmega \cap \{z_3=0\}$(i.e. the unit disc in ${\mathbb {C}}(z_1)$). We denote by $A^\infty (\varOmega ), A^\infty (\varOmega _0)$ the subspace of $C^\infty ({\overline{\varOmega }}), C^\infty ({\overline{\varOmega }}_0)$ given by the functions which are holomorphic in the interior of $\varOmega , \varOmega _0$.

### Proposition 8.1

The restriction map $A^\infty (\varOmega )\rightarrow A^\infty (\varOmega _0)$ is surjective. More precisely, for all $f\in A^\infty (\varOmega _0)$ there is ${\widetilde{f}}\in A^\infty (\varOmega )$ such that ${\widetilde{f}}|_{\varOmega _0}=f$ and $\frac{\partial ^k {\widetilde{f}}}{\partial z_3^k}|_{\varOmega _0}=0$ for all $k\ge 1$.

To achieve the proof of the Proposition, we modify the construction in [6], and sometimes refer to lemmas in there without further mention. First, we need to prove an estimate which will be useful later:

### Lemma 8.1

Fixed $r>0$, we have

$$\begin{aligned} \frac{1}{2}\log (j^2)\le \frac{1}{\sin (1/j^{r+2})}(1 - (j^{r}\sin (1/j^{r+2}))^{\frac{1}{j^{r+2}}})\le 2\log (2j^2) \end{aligned}$$

for all large enough $j\in {\mathbb {N}}$.

### Proof

Put $x_j= 1 - (j^{r}\sin (1/j^{r+2}))^{\frac{1}{j^{r+2}}}$; then $x_j>0$ and

$$\begin{aligned} (1-x_j)^{j^{r+ 2}}=j^{r}\sin (1/j^{r+2}). \end{aligned}$$

Moreover, since $(j^{r}\sin (1/j^{r+2}))^{\frac{1}{j^{r+2}}}\ge (1/2j^2)^{\frac{1}{j^{r+2}}}\rightarrow 1$ as $j\rightarrow \infty$, we have $x_j\rightarrow 0$ as $j\rightarrow \infty$. From the expression above we get

$$\begin{aligned} \log (1-x_j)=\frac{1}{j^{r+2}} \log (j^{r}\sin (1/j^{r+2})); \end{aligned}$$

since for *j* large enough we have $-2x_j\le \log (1-x_j)\le -x_j$ and $\frac{1}{2j^{r+2}}\le \sin (1/j^{r+2}) \le \frac{1}{j^{r+2}}$, we can write

$$\begin{aligned} -x_j\ge \frac{\log (1/2j^2) }{j^{r+2}} \Rightarrow x_j\le \frac{\log (2j^2) }{j^{r+2}}, \ -x_j\le \frac{\log (1/j^2) }{2j^{r+2}} \Rightarrow x_j\ge \frac{\log (j^2) }{2j^{r+2}} \end{aligned}$$

for large *j*. The conclusion follows from these inequalities and again from the fact that $\frac{1}{2j^{r+2}}\le \sin (1/j^{r+2}) \le \frac{1}{j^{r+2}}$ for large enough *j*.

Fix an increasing sequence $\{m_\ell \}$ of positive integers such that $m_\ell \ge e^\ell$. Define sequences of functions $\{\psi _j\}$, $\{\xi _j\}$, $\{\varphi _j\}$ by putting

$$\begin{aligned} \psi _j(z_1,z_3)=-\frac{\lambda (1/j^{k_0\ell + 2})}{z^{\ell /j^{k_0\ell +2} }}+B(1/j^{k_0\ell +2}),\ \ \xi _j=e^{\psi _j}, \ \ \varphi _j=e^{-\xi _j}. \end{aligned}$$

for all $m_{\ell }\le j< m_{\ell +1}$, where $B(y)=1/\sin (y)$ and $\lambda (y)= B(y)^{1-y}$. Note that $\psi _j$ is well-defined on $\varOmega {\setminus } \varOmega _0$, and ${\mathrm Re}\, \psi _j(z_1,z_3)\rightarrow -\infty$ as $z_3\rightarrow 0$. Furthermore the function $\varphi _j$ extends continuously to ${\overline{\varOmega }}$ and $\varphi _j\equiv 1$ on $\varOmega _0$. Put $D_j=\{(z_1,z_3)\in \varOmega : |z_3|\le 1/j^{k_0})\}$, and fix $p\in \varOmega {\setminus } D_j$. With the same computations as in Lemma 4.1 (choosing $A_\beta =j^2$) we have $|{\mathrm Im}\, \psi _{j}(p)|\le \frac{1}{\cos (\ell /j^{2\ell + 2})}=:d_j$ for $m_{\ell }\le j< m_{\ell +1}$ and

$$\begin{aligned}&{\mathrm Re}\, \psi _{j}(p)\ge B\left( 1/j^{k_0\ell +2}\right) \left( 1-\left( \frac{(j^{k_0})^{\ell }}{B\left( 1/j^{k_0\ell +2}\right) }\right) ^{\frac{1}{j^{k_0\ell +2}}}\right) =\\&\quad =\frac{1}{\sin (1/j^{k_0\ell +2})}(1 - (j^{k_0\ell }\sin (1/j^{k_0\ell +2}))^{\frac{1}{j^{k_0\ell +2}}})\ge \log (j^2)/2 = \log (j) \end{aligned}$$

by Lemma 8.1. Choose $1<d<\pi /2$ such that $d\ge d_j$ for all $j\in {\mathbb {N}}$ large enough (indeed $d_j\rightarrow 1$ as $j\rightarrow \infty$). From the expression above follows that

$$\begin{aligned} {\mathrm Re}\, \xi _j(p)\ge |\xi _j(p)|\cos (d_j)=e^{{\mathrm Re}\, \psi _{j}(p)}\cos (d_j)\ge j\cos (d_j) \ge j\cos (d) \end{aligned}$$

and thus

$$\begin{aligned} |\varphi _j(p)|=e^{-{\mathrm Re}\, \xi _j(p)}\le e^{-j\cos (d)}. \end{aligned}$$

On the other hand we have $|\varphi _j(p)|\le e$ for all $p\in D_j$ (same proof as in Lemma 4.2).

### Lemma 8.2

For all $p=(z_1,z_3)\in \varOmega$ we have $|\varphi _j(p)|\le \frac{e^2}{(1+\root k_0 \of {|z_3|})^j}$.

### Proof

Suppose first that $p\in D_j$, i.e. $|z_3|\le 1/j^{k_0}$. Then $\frac{e^2}{(1+\root k_0 \of {|z_3|})^j}\ge \frac{e^2}{(1+1/j)^j}\ge e \ge |\varphi _j(p)|$. If instead $p\in \varOmega {\setminus } D_j$ we can write $\frac{e^2}{(1+\root k_0 \of {|z_3|})^j}\ge \frac{1}{(1+\root k_0 \of {\tau })^j}= \frac{1}{(e^{cos(d)})^j}\ge |\varphi _j(p)|$ if $\tau >0$ is small enough.

The next statement is an immediate consequence of the chain rule.

### Lemma 8.3

Fix $k\in {\mathbb {N}}$. There is a polynomial $P_k(X_1,X_2,\ldots ,X_k)$ such that

$$\begin{aligned} \frac{\partial ^k \varphi _j}{\partial z_3^k}=\varphi _jP_k\left( \frac{\partial \xi _j}{\partial z_3}, \frac{\partial ^2 \xi _j}{\partial z_3^2},\ldots ,\frac{\partial ^k \xi _j}{\partial z_3^k}\right) \end{aligned}$$

for all $j\in {\mathbb {N}}$. Furthermore, $P_k$ is weighted homogeneous of degree *k* (where the variable $X_j$ has weight *j*).

Thus, to obtain an estimate for $\frac{\partial ^k \varphi _j}{\partial z_3^k}$ we need to give one for $\frac{\partial ^h \xi _j}{\partial z_3^h}$, $h\le k$. In the next lemma we show that $|\frac{\partial ^h \xi _j}{\partial z_3^h}(p)|$ grows as a polynomial in *j* if $p\in \varOmega \cap D_j$, while if $p\in \varOmega {\setminus } D_j$ its growth is compensated by the exponential decay of $|\varphi _j(p)|$, resulting in the following statement:

### Lemma 8.4

Let $k\in {\mathbb {N}}$, $k\ge 1$. There exist $N_k>0$, $\tau '>0$ such that

$$\begin{aligned} \left| \frac{\partial ^k \varphi _j}{\partial z_3^k}(p) \right| \le N_k j^{3k_0k}\frac{1}{(1+\root k_0 \of {|z_3|})^j} \end{aligned}$$

for all $p=(z_1,z_3)\in \varOmega$ with $|z_3|\le \tau '$ and all $j\in {\mathbb {N}}$.

### Proof

In the following we always consider $\ell ,j\in {\mathbb {N}}$ such that $m_{\ell }\le j< m_{\ell +1}$, and fix $h\in {\mathbb {N}}$. Moreover we put $y_j=1/j^{k_0\ell +2}$. The following expression for $\frac{\partial ^h \xi _j}{\partial z_3^h}$ can be checked inductively:

$$\begin{aligned} \frac{\partial ^h \xi _j}{\partial z_3^h}=\xi _j\sum _{a=1}^h \beta _{a,j}\frac{\ell ^a}{z_3^{a\ell y_j + h}} \end{aligned}$$

where $\beta _{a,j}$ is bounded in *j* for all $1\le a\le h$. Thus we have

$$\begin{aligned} \left| \frac{\partial ^h \xi _j}{\partial z_3^h} \right| \le |\xi _j| \sum _{a=1}^h |\beta _{a,j}| \frac{\ell ^a}{|z_3|^{a\ell y_j + h}}\le C_h \ell ^h |\xi _j| \frac{1}{|z_3|^{h\ell y_j + h}} \end{aligned}$$

for some constant $C_h>0$ (independent of *j*). Taking in account the definition of $\xi _j$, we can write

$$\begin{aligned} \left| \frac{\partial ^h \xi _j}{\partial z_3^h} \right| \le C_h\ell ^h\frac{1}{|z_3|^{h\ell y_j + h}} e^{-{\mathrm Re}\,\frac{\lambda (y_j)}{z_3^{\ell y_j}}} e^{B(y_j)}\\ \le C_h\ell ^h\frac{1}{|z_3|^{h\ell y_j + h}} e^{-\frac{\lambda (y_j)\cos (\pi \ell y_j)}{|z_3|^{\ell y_j}}} e^{B(y_j)}. \end{aligned}$$

Define the function $\kappa :{\mathbb {R}}^+\rightarrow {\mathbb {R}}^+$ as

$$\begin{aligned} \kappa (r)=\frac{1}{r^{h\ell y_j + h}} e^{-\frac{\lambda (y_j)\cos (\pi \ell y_j)}{r^{\ell y_j}}}; \end{aligned}$$

clearly $\kappa (r)\rightarrow 0$ as $r\rightarrow 0^+$ and as $r\rightarrow +\infty$. Computing the first derivative

$$\begin{aligned} \kappa '(r)=\left( -\frac{h\ell y_j + h}{r^{h\ell y_j+h+1}}+\frac{\ell y_j \lambda (y_j)\cos (\pi \ell y_j)}{r^{(h+1)\ell y_j + h+1}}\right) e^{-\frac{\lambda (y_j)\cos (\pi \ell y_j)}{r^{\ell y_j}}} \end{aligned}$$

we see that it vanishes only at

$$\begin{aligned} {\widetilde{r}}=\left( \frac{\ell y_j \lambda (y_j)\cos (\pi \ell y_j)}{h\ell y_j + h}\right) ^{1/\ell y_j} \end{aligned}$$

hence $\kappa$ is increasing for $0\le r<{\widetilde{r}}$ and decreasing for $r>{\widetilde{r}}$. Furthermore

$$\begin{aligned} (y_j\lambda (y_j))^{1/\ell {y_j}}= & {} \left( \frac{y_j\sin (y_j)^{y_j}}{\sin (y_j)}\right) ^{1/\ell y_j}=\left( \frac{y_j}{\sin (y_j)}\right) ^{1/\ell y_j}\sin (y_j)^{1/\ell } \\= & {} \left( \frac{1}{(1-y_j^2/6+O(y_j^4))^{1/y_j}}\sin (y_j) \right) ^{1/\ell } \end{aligned}$$

and

$$\begin{aligned} \cos (\pi \ell y_j)^{1/\ell y_j}=(1-(\pi \ell y_j)^2/2 + O((\pi \ell y_j)^4))^{1/\ell y_j} \end{aligned}$$

are bounded (above and below) independently of *j*, so that for some $K>0$ we can write

$$\begin{aligned} \begin{aligned} {\widetilde{r}}&\ge K \left( \frac{\ell }{h\ell y_j + h}\right) ^{1/\ell y_j} =K\left( \frac{\ell }{h}\right) ^{j^{k_0\ell +2}/\ell }\left( \frac{1}{1+ \ell /j^{k_0\ell +2} }\right) ^{j^{k_0\ell +2}/\ell } \\&\ge \frac{K}{e}\left( \frac{\ell }{h}\right) ^{j^{k_0\ell +2}/\ell }. \end{aligned} \end{aligned}$$

If $h\le \ell$ we obtain ${\widetilde{r}}\ge \frac{K}{e}$. In fact, if $h<\ell$ we have ${\widetilde{r}}\rightarrow \infty$ as $j\rightarrow \infty$, so we can assume ${\widetilde{r}}\ge \tau$. Thus the function $\kappa$ is increasing on the interval $[0,\tau ]$.

Let $p\in \varOmega \cap D_j$, $p=(z_1,z_3)$. Since $|z_3|\le 1/j^{k_0}$ we have

$$\begin{aligned} \begin{aligned} \left| \frac{\partial ^h \xi _j}{\partial z_3^h}(p) \right|&\le C_h\ell ^h \kappa (|z_3|) e^{B(1/j^{k_0\ell +2})} \\&\le C_h\ell ^h \kappa (1/j^{k_0}) e^{B(1/j^{k_0\ell +2})} \\&\le C_h\ell ^h j^{k_0h(1+\ell /j^{k_0\ell +2})} \frac{\exp \left( B(1/j^{k_0\ell +2})\right) }{\exp \left( j^{k_0\ell /j^{k_0\ell +2}}\lambda (1/j^{k_0\ell +2})\cos (\pi \ell /j^{k_0\ell +2})\right) } \\&= C_h\ell ^h j^{k_0h(1+\ell /j^{k_0\ell +2})}\exp (\alpha _j). \end{aligned} \end{aligned}$$

We can rewrite the argument of the exponential as follows:

$$\begin{aligned} \begin{aligned} \alpha _j&= \frac{1}{\sin (1/j^{k_0\ell +2})}\left( 1 - \cos (\pi \ell /j^{k_0\ell +2})(j^{k_0\ell }\sin (1/j^{k_0\ell +2}))^{\frac{1}{j^{k_0\ell +2}}}\right) \\&= \frac{1}{\sin (1/j^{k_0\ell +2})}\left( 1 - (j^{k_0\ell }\sin (1/j^{k_0\ell +2}))^{\frac{1}{j^{k_0\ell +2}}}\right) \\&\qquad + \frac{1-\cos (\pi \ell /j^{k_0\ell + 2})}{\sin (1/j^{k_0\ell +2})}(j^{k_0\ell }\sin (1/j^{k_0\ell +2}))^{\frac{1}{j^{k_0\ell +2}}}. \end{aligned} \end{aligned}$$

The second summand in the expression above is bounded (in fact it can be seen to be $O(\ell ^2/j^{k_0\ell +2})$), while the first one is estimated by $2\log (2j^2)$ by Lemma 8.1. We deduce that

$$\begin{aligned} \left| \frac{\partial ^h \xi _j}{\partial z_3^h}(p) \right| \le C_h\ell ^h j^{k_0h(1+\ell /j^{k_0\ell +2})}\exp (\log (j^4)+O(1))\le C'_h j^{3k_0h} \end{aligned}$$

for a large enough $C'_h>0$ (here we are using the fact that $\ell \le \log (j)$ by the choice of $m_\ell$).

The estimate above, together with Lemma 8.3, show that there exists $N'_k>0$ such that

8.1 $$\begin{aligned} \left| \frac{\partial ^k \varphi _j}{\partial z_3^k}(p) \right| \le N'_k j^{3k_0k} \end{aligned}$$

for all $$p\in \varOmega \cap D_j$$.

Consider now $p\in \varOmega {\setminus } D_j$, $p=(z_1,z_3)$. Since $|z_3|\ge 1/j^{k_0}$ we have

$$\begin{aligned} \left| \frac{\partial ^h \xi _j}{\partial z_3^h}(p) \right| \le C_h\ell ^h\frac{1}{|z_3|^{h\ell /j^{k_0\ell +2} + h}}|\xi _j(p)| \le C_h\ell ^h j^{k_0h(1+\ell /j^{k_0\ell +2})} |\xi _j(p)|\\\le C_h(\log (j))^h j^{k_0h(1+\ell /j^{k_0\ell +2})} |{\mathrm Re}\, \xi _j| \cos (d_j)\le C''_h j^{2k_0h}(-\log (|\varphi _j(p)|)). \end{aligned}$$

As before, using Lemma 8.3 we get that there exists $N''_k>0$ such that

$$\begin{aligned} \left| \frac{\partial ^k \varphi _j}{\partial z_3^k}(p) \right| \le N''_k j^{2k_0k} |\varphi _j(p)|(-\log (|\varphi _j(p)|))^k \end{aligned}$$

for all $p\in \varOmega {\setminus } D_j$. Since $|\varphi _j(p)|\le e^{-j\cos (d)}\rightarrow 0$ as $j\rightarrow \infty$ we have that

$$\begin{aligned} (-\log (|\varphi _j(p)|))^k\le 1/\sqrt{|\varphi _j(p)|} \end{aligned}$$

for all $p\in \varOmega {\setminus } D_j$ and all large enough *j*, and thus

8.2 $$\begin{aligned} \left| \frac{\partial ^k \varphi _j}{\partial z_3^k}(p) \right| \le N''_k j^{2k_0k} \sqrt{|\varphi _j(p)|}\le N''_k j^{2k_0k}e^{-j\cos (d)/2}. \end{aligned}$$

Using that $1/(1+\root k_0 \of {|z_3|})^j\ge 1/e$ if $|z_3|\le 1/j^{k_0}$ and $1/(1+\root k_0 \of {|z_3|})^j \ge e^{-j\cos (d)/2}$ if $|z_3|\le \tau '$ small enough, we can put together (8.1) and (8.2) as in Lemma 8.2 to conclude that there exists $N_k>0$ such that

$$\begin{aligned} \left| \frac{\partial ^k \varphi _j}{\partial z_3^k}(p) \right| \le N_k j^{3k_0k} \frac{1}{(1+\root k_0 \of {|z_3|})^j} \end{aligned}$$

for all $$p\in \varOmega $$, $$j\in {\mathbb {N}}$$.

### Proof of Proposition 8.1:

For $t>0$, define the dilation $\psi _t:{\mathbb {C}}^2\rightarrow {\mathbb {C}}^2$ as $\psi _t(z_1,z_3)=(z_1,tz_3)$, and let $\varOmega ^t=\psi _t^{-1}(\varOmega )$. We have

$$\begin{aligned} \varOmega ^t= \{(z_1,z_3)\in {\mathbb {C}}^2: x_3\ge 0, \ |z_3|<\tau /t, \ |z_1|<1+\root k_0 \of {t}\root k_0 \of {x_3}\} \end{aligned}$$

so that $\varOmega ^t\cap \{z_3=0\}=\varOmega _0$ and $\varOmega ^t\cap \{|z_3|<\tau \}\subset \varOmega$ if $t<1$. For a given $f\in A^\infty (\varOmega _0)$, we will construct ${\widetilde{f}}$, holomorphic in the interior of $\varOmega$, such that $\frac{\partial ^{h+k} {\widetilde{f}}}{\partial z_1^h\partial z_3^k}$ extends continuously to $\varOmega _0$ for all $h,k\ge 0$. Then it is clear that ${\widetilde{f}}|_{ \varOmega ^t}\in A^\infty (\varOmega ^t)$, and thus ${\widetilde{f}}\circ \psi _t^{-1}\in A^\infty (\varOmega )$; furthermore ${\widetilde{f}}\circ \psi _t^{-1}|_{\varOmega _0}=f$ since $\psi _t$ is the identity on $\varOmega _0$.

Let $f\in A^\infty (\varOmega _0)$, $f(z_1)= \sum _{j=0}^\infty a_j z_1^j$. Since *f* is smooth up to $b\varOmega _0$ the sequence $a_j$ goes to 0 faster than any polynomial, that is for all $k\in {\mathbb {N}}$ there is $A_k>0$ such that $|a_j|\le A_k/j^k$ for all $j\ge 1$.

We define now ${\widetilde{f}}(z_1,z_3)= \sum _j a_j z_1^j \varphi _j(z_3)$. By Lemma 8.2 follows that the series $\sum _j a_j z_1^j \varphi _j$ converges uniformly on compact sets of the interior of $\varOmega$, hence ${\widetilde{f}}$ is a well-defined holomorphic function in the interior of $\varOmega$. We will show now that, for all $k\ge 1$, $\sup _{\varOmega _c}|\frac{\partial ^k {\widetilde{f}}}{\partial z_3^k}| \rightarrow 0$ as $c\rightarrow 0$, where $\varOmega _c=\varOmega \cap \{z_3=c\}$. This will imply that ${\widetilde{f}}$ (as well as $\frac{\partial ^k {\widetilde{f}}}{\partial z_3^k}$) extends continuously to $\varOmega _0$, and ${\widetilde{f}}|_{\varOmega _0}=f$. The same argument, applied to $\frac{\partial ^h {\widetilde{f}}}{\partial z_1^h}$, proves that $\frac{\partial ^{h+k} {\widetilde{f}}}{\partial z_1^h\partial z_3^k}$ extends continuously to $\varOmega _0$.

Fix then $k\in {\mathbb {N}}$, and let $A_{3k_0k+2}>0$ such that $|a_j|\le A_{3k_0k+2}/j^{3k_0k+2}$ for all $j\in {\mathbb {N}}$. Given $\epsilon >0$, let $j_0\in {\mathbb {N}}$ such that $N_k A_{3k_0k+2}\sum _{j> j_0} \frac{1}{j^2}<\epsilon$. For any $p=(z_1,z_3)\in \varOmega$ we get

$$\begin{aligned} \begin{aligned} \left| \frac{\partial ^k{\widetilde{f}}}{\partial z_3^k}(p)\right|&=\left| \sum _{j\le j_0}a_j z_1^j \frac{\partial ^k\varphi _j}{\partial z_3^k}(z_3) + \sum _{j>j_0}a_j z_1^j \frac{\partial ^k\varphi _j}{\partial z_3^k}(z_3)\right| \\&\le \left| \sum _{j\le j_0}a_j z_1^j \frac{\partial ^k\varphi _j}{\partial z_3^k}(z_3)\right| + \sum _{j>j_0}|a_j| |z_1|^j \left| \frac{\partial ^k\varphi _j}{\partial z_3^k}(z_3) \right| \\&\le \left| \sum _{j\le j_0}a_j z_1^j \frac{\partial ^k\varphi _j}{\partial z_3^k}(z_3)\right| + \sum _{j>j_0}\frac{A_{3k_0k+2}}{j^{3k_0k+2}}N_kj^{3k_0k}\left( \frac{|z_1|}{1+\root k_0 \of {|z_3|}}\right) ^j \\&\le \left| \sum _{j\le j_0}a_j z_1^j \frac{\partial ^k\varphi _j}{\partial z_3^k}(z_3)\right| + \epsilon \end{aligned} \end{aligned}$$

where we used Lemma 8.4 and the fact that $|z_1|\le (1+\root k_0 \of {x_3})\le (1+\root k_0 \of {|z_3|})$. Since $\sum _{j\le j_0}a_j z_1^j \frac{\partial ^k\varphi _j}{\partial z_3^k}(z_3)$ is a finite sum and $\varphi _j$ is flat at 0 for all *j*, we conclude that $\left| \frac{\partial ^k{\widetilde{f}}}{\partial z_3^k}(p)\right| <2\epsilon$ for $|z_3|$ small enough.

### Corollary 8.1

Define $\Gamma \subset {\mathbb {C}}^3(z_1,z_2,z_3)$ as the set

$$\begin{aligned} \Gamma = \{(z_1,z_2,z_3)\in {\mathbb {C}}^3: x_3\ge 0, \ |z_3|<\tau , \ |z_1|^2+|z_2|^2<1+\root k_0 \of {x_3}\} \end{aligned}$$

and let $\Gamma _0=\Gamma \cap \{z_3=0\}$ be the unit ball in ${\mathbb {C}}^2$. Then the restriction map $A^\infty (\Gamma )\rightarrow A^\infty (\Gamma _0)$ is surjective. More precisely, for all $f\in A^\infty (\Gamma _0)$ there is ${\widetilde{f}}\in A^\infty (\Gamma )$ such that ${\widetilde{f}}|_{\Gamma _0}=f$ and $\frac{\partial ^k {\widetilde{f}}}{\partial z_3^k}|_{\Gamma _0}=0$ for all $k\ge 1$.

### Proof

Given $f\in A^\infty (\Gamma _0)$, we can apply the construction of Proposition 8.1 on the slices $\Gamma \cap \{\alpha z_1=\beta z_2\}$ to define an extension ${\widetilde{f}}$ of *f* to $\Gamma$, holomorphic on each slice. Since the sequence of “cut off” functions $\varphi _j$ is independent of $\alpha , \beta$, ${\widetilde{f}}$ is in fact globally holomorphic in $(z_1,z_2,z_3)$.

### Corollary 8.2

Define $\varSigma ''\subset {\mathbb {C}}^3(z_1,z_2,z_3)$ as the set

$$\begin{aligned} \varSigma ''=\{(z_1,z_2,z_3)\in {\mathbb {C}}^3: x_3\ge 0, \ \ x_1\ge x_2^2-\root k_0 \of {x_3}\} \end{aligned}$$

and let (*S*, 0) be a germ of smooth real hypersurface of ${\mathbb {C}}^3$ such that $0\in S$ and $S\subset \varSigma ''$. Furthermore let $S_0=S\cap \{z_3=0\}$. Then for any formal series $\sigma =\sum _{j_1,j_2} a_{j_1j_2}z_1^{j_1} z_2^{j_2}$ there is a (germ of a) solution $g\in {\mathfrak {S}}(S)$ whose Taylor series at 0 is given by $\sigma$ and $\frac{\partial ^k g}{\partial z_3^k}|_{S_0}=0$ for all $k\ge 1$.

### Proof

Define the Cayley transformation $\varPhi :{\mathbb {C}}^3{\setminus }\{z_1=1\}\rightarrow {\mathbb {C}}^3$ as

$$\begin{aligned} \varPhi (z_1,z_2,z_3)= \left( \frac{1+z_1}{1-z_1}, \frac{z_2}{1-z_1},z_3\right) ; \end{aligned}$$

we have that $\varPhi$ maps $\Gamma '$ to $\varSigma ''$, where

$$\begin{aligned} \Gamma ' = \{(z_1,z_2,z_3)\in {\mathbb {C}}^3: x_3\ge 0, \ |z_3|<\tau , \ |z_1|^2+|z_2|^2<1+|1-z_1|^2\root k_0 \of {x_3}\} \end{aligned}$$

on the other hand, since $|1-z_1|^2$ is bounded we have (locally) $\Gamma '\subset \Gamma ''$ with

$$\begin{aligned} \Gamma '' = \{(z_1,z_2,z_3)\in {\mathbb {C}}^3: x_3\ge 0, \ |z_3|<\tau , \ |z_1|^2+|z_2|^2<1+C\root k_0 \of {x_3}\} \end{aligned}$$

for some large enough $C>0$. However $\Gamma ''$ is biholomorphic to the set $\Gamma$ of Corollary 8.1 via a rescaling of the $z_3$ coordinate, so the conclusion of Corollary 8.1 holds for $\Gamma ''$. Since $\varPhi (-1,0,0) = (0,0,0)$ we can consider $\sigma '=\sigma \circ \varPhi$ as a formal power series centered at the point $p_0=(-1,0,0)\in \Gamma ''_0$. Since $\psi =-i(z_1+1)$ is a (global) peak function of finite order for $\Gamma ''_0$ at $p_0$, there exists a smooth CR solution $f\in {\mathfrak {S}}(\Gamma ''_0)$ whose Taylor expansion at $p_0$ is $\sigma '$. By Corollary 8.1 there exists ${\widetilde{f}}\in A^\infty (\Gamma '')$ such that ${\widetilde{f}}|_{\Gamma ''_0}=f$ and $\frac{\partial ^k {\widetilde{f}}}{\partial z_3^k}|_{\Gamma ''_0}=0$ for all $k\ge 1$. Putting $g={\widetilde{f}} \circ \varPhi ^{-1}$, we have that *g* is defined on a neighborhood of 0 in $\varSigma ''$ and smooth up to the boundary. By construction $g|_S$ satisfies the requirements of the Corollary.

Consider now for $\ell \ge 0$ the hypersurface

$$\begin{aligned} \varSigma =\{x_3=(x_1^{2\ell +1}-x_2^2)^2 \}\subset {\mathbb {C}}^3 \end{aligned}$$

and put $$\varSigma _0=\varSigma \cap \{z_3=0\}$$.

Using the notation of section 3 with $m=3, p=2$, we consider the partial Borel maps

$$\begin{aligned} {\mathfrak {b}}_1 :{\mathfrak {S}}^{(1)}_0\rightarrow {{\mathbb {C}}}\llbracket z_1,z_2 \rrbracket , \,\,{\mathfrak {b}}_2 :{\mathfrak {S}}_0^{(2)} \rightarrow {{\mathbb {C}}}\llbracket z_{3} \rrbracket . \end{aligned}$$

We have that ${\mathfrak {b}}_2$ is surjective because ${z_3}_{|\varSigma }=(x_1^{2\ell +1}-x_2^2)^2+iy_3$ is a peak function at 0, which implies that the corank 1 structure induced on $\varSigma$ by the function $z_3$ satisfies the Borel property.

In view of Theorem 4.1, the first claim of Theorem 8.3 is proved if ${\mathfrak {b}}_1$ is also surjective. This is the content of the following statement:

### Proposition 8.2

Let $\sum _{j_1,j_2} a_{j_1j_2}z_1^{j_1} z_2^{j_2}$ be any formal series in $(z_1,z_2)$. Then there is a neighborhood *U* of 0 in $\varSigma$ and a solution $g\in {\mathfrak {S}}(U)$ such that

- the Taylor expansion of *g* at 0 is given by $\sum _{j_1,j_2} a_{j_1j_2}z_1^{j_1} z_2^{j_2}$;
- $$\frac{\partial ^k g}{\partial z_3^k}|_{\varSigma _0}=0$$ for all $$k\ge 1$$.

### Proof

Define the domains

$$\begin{aligned} \varSigma '= & {} \{(z_1,z_2,z_3)\in {\mathbb {C}}^3: x_3\ge 0, \ \ x_1\ge \root 2\ell +1 \of {x_2^2-\sqrt{x_3}}\},\\ \varSigma ''= & {} \{(z_1,z_2,z_3)\in {\mathbb {C}}^3: x_3\ge 0, \ \ x_1\ge x_2^2-2\root 4\ell +2 \of {x_3}\}. \end{aligned}$$

It is clear that $\varSigma \subset \varSigma '$; we claim that, if $\epsilon >0$ is small enough, $\varSigma '\cap B_\epsilon (0)\subset \varSigma ''\cap B_\epsilon (0)$ (where $B_\epsilon (0)\subset {\mathbb {C}}^3$ is the ball of radius $\epsilon$ centered at 0). Indeed, for (small) fixed $x_3\ge 0$ consider the function $\gamma :{\mathbb {R}}^+\rightarrow {\mathbb {R}}$

$$\begin{aligned} \gamma (t)=\root 2\ell +1 \of {\sqrt{x_3}-t} - (2\root 4\ell +2 \of {x_3} - t). \end{aligned}$$

Looking at the interval $[0,2\root 4\ell +2 \of {x_3}]$ we note that $\gamma (0)=-\root 4\ell +2 \of {x_3}\le 0$ and moreover $\gamma (2\root 4\ell +2 \of {x_3})=\root 2\ell +1 \of {\sqrt{x_3}-2\root 4\ell +2 \of {x_3}}\le \root 2\ell +1 \of {\sqrt{x_3}-2\sqrt{x_3}}= -\root 4\ell +2 \of {x_3}\le 0$. On the other hand we have

$$\begin{aligned} \gamma '(t)=-\frac{1}{2\ell +1}\cdot \frac{1}{(\sqrt{x_3}-t)^{2\ell /(2\ell +1)}}+1 \end{aligned}$$

hence $\gamma '$ vanishes exactly at $t=\sqrt{x_3}\pm \frac{1}{\root 2\ell \of {(2\ell +1)^{2\ell +1}}}$. If $x_3$ is small enough, neither of these values lies in the interval $[0,2\root 4\ell +2 \of {x_3}]$, showing that $\gamma$ is monotone on that interval. Since $\gamma (0)\le 0$ and $\gamma (2\root 4\ell +2 \of {x_3})\le 0$ we must have $\gamma \le 0$ on $[0,2\root 4\ell +2 \of {x_3}]$, i.e.

$$\begin{aligned} \root 2\ell +1 \of {x_2^2-\sqrt{x_3}}\ge x_2^2-2\root 4\ell +2 \of {x_3} \ \hbox { for } 0\le x_2^2\le 2\root 4\ell +2 \of {x_3}. \end{aligned}$$

If instead $\sqrt{x_3}\le x_2^2\le 1$ we have $0\le x_2^2-\sqrt{x_3}\le 1$, so we can write (for small $x_3$)

$$\begin{aligned} \root 2\ell +1 \of {x_2^2-\sqrt{x_3}}\ge x_2^2-\sqrt{x_3}\ge x_2^2-2\root 4\ell +2 \of {x_3} \ \hbox { for } \sqrt{x_3}\le x_2^2\le 1. \end{aligned}$$

Since $\sqrt{x_3}\le \root 4\ell +2 \of {x_3}$ we conclude that $\root 2\ell +1 \of {x_2^2-\sqrt{x_3}}\ge x_2^2-2\root 4\ell +2 \of {x_3}$ for $x_3$ small enough and $-1\le x_2\le 1$, which proves the claimed inclusion $\varSigma '\cap B_\epsilon (0)\subset \varSigma ''\cap B_\epsilon (0)$.

Using a suitable change of coordinates we can map $\varSigma ''$ biholomorphically to the domain $\{x_1\ge |z_2|^2-\root 4\ell +2 \of {x_3}\}$, so that $\varSigma ''_0$ is a one-sided neighborhood of the Lewy hypersurface $\{x_1=|z_2|^2\}$. We denote again by $\sigma =\sum _{j_1,j_2} a_{j_1j_2}z_1^{j_1} z_2^{j_2}$ the formal series obtained by transforming the one in the statement through this coordinate change. The conclusion of the Proposition follows then by applying Corollary 8.2 with $S=\varSigma$ and $k_0=4\ell +2$.

By using the methods above, we can deduce directly the following (apparently more general) consequence:

### Theorem 8.4

With the notation of Theorem 8.1, suppose that $n=2$ and $\phi (t)=(f(t_1,t_2))^2$ where the differential of *f* does not vanish at 0 and the domain $\{f(t_1,t_2)>0\}$ is strictly convex (or concave) around 0. Then the Borel map is surjective.

### Proof

Let us consider the tube manifold $\varSigma = \{x_3=(f(x_1,x_2))^2\}$. Up to a linear change of coordinates, we can suppose that the tangent line of $\{f=0\}$ at 0 is $\frac{\partial }{\partial x_1}$ and $\{f>0\}$ is (locally) strictly convex. Then it is easy to show that there exists $C>0$ such that $f(x_1,x_2)\ge x_1-Cx_2^2$ for all $x_1,x_2$ around 0. This implies that $\varSigma$ is locally contained in the domain

$$\begin{aligned} \{(z_1,z_2,z_3)\in {\mathbb {C}}^3: x_3\ge 0, \ \ x_1\ge Cx_2^2-\sqrt{x_3}\}. \end{aligned}$$

From Corollary 8.2 follows that the partial Borel map ${\mathfrak {b}}_1$ is surjective, which implies the Borel property just as in the proof of the first claim in Theorem 8.3.

## The structure of the maximal ideal of ${\mathfrak {S}}_p$

**A.** In most of this section we shall assume that the locally integrable structure ${\mathcal {V}}$ over $\varOmega$ satisfies condition (${\mathfrak {B}}$) at $p\in \varOmega$ (cf. Section 6B).

According to ([3], proof of Theorem 6.1) we can assert the following: *p* is the center of a smooth coordinate system $$(x_1,\ldots ,x_m,$$$$t_1,\ldots ,t_n)$$, which can be assumed defined in a product $$U=B\times \varTheta $$, where *B* (respectively $$\varTheta $$) is an open ball centered at the origin in $${{\mathbb {R}}}_x^m$$ (respectively $${{\mathbb {R}}}_t^n$$), over which there is defined a smooth, real vector-valued function $$\varPhi (x,t)=(\varPhi _1(x,t),\dots ,\varPhi _m(x,t))$$ satisfying $$\varPhi (0,0)=0$$, $$D_x\varPhi (0,0)=0$$, in such a way that the differential of the functions

$$\begin{aligned} Z_k(x,t)=x_k+i\varPhi _k(x,t),\qquad k=1,\ldots , m, \end{aligned}$$

span ${\mathcal {V}}^\perp$ over *U*. Contracting *U* even more around the origin we may achieve:

1. $$\hbox {d}Z_m(0,0)\in T^0_{(0,0)}$$ and $$\arg Z_m\ne -\pi $$ in *U*;
2. There are constants $C,M>0$ so that $(|Z_1|+\ldots +|Z_{m-1}|)^M\le C|Z_m|$ in *U*.

As before we can consider the corresponding vector fields ${\mathrm {L}}_j$, ${\mathrm {M}}_k$ satisfying the standard orthogonality conditions.

**B.**${\mathfrak {S}}_0$ is a commutative local ring with maximal ideal

$$\begin{aligned} {\mathfrak {m}}= \{u\in {\mathfrak {S}}_0: \, u(0)=0\}. \end{aligned}$$

Our goal now is to give sufficient conditions in order to insure that ${\mathfrak {m}}$ is a finitely generated ${\mathfrak {S}}_0$-module. This is of course true when ${\mathcal {V}}$ is hypocomplex at the origin. On the other hand we also have the following result:

### Theorem 9.1

Assume that ${\mathcal {V}}$ satisfies condition (${\mathfrak {B}}$) at the origin. If either ${\mathcal {V}}$ is minimal at the origin or if ${\mathcal {V}}$ is a real-analytic locally integrable structure then the following holds: if $W_1,\ldots W_m\in {\mathfrak {m}}$ are such that $\hbox {d}W_1(0),\ldots , \hbox {d}W_m(0)$ are linearly independent then

$$\begin{aligned} {\mathfrak {m}}= \langle W_1,\ldots W_m\rangle \end{aligned}$$

as a $${\mathfrak {S}}_0$$-module

We start by proving:

### Lemma 9.1

If ${\mathcal {V}}$ satisfies condition (${\mathfrak {B}}$) given $u\in {\mathfrak {m}}$ there are $v_j\in {\mathfrak {S}}_0$ such that

$$\begin{aligned} u - \sum _{j=1}^m v_j Z_j \in \ker {\mathfrak {b}}. \end{aligned}$$

### Proof

Since $u(0)=0$ we can write ${\mathfrak {b}}(u)=\sum _{j=1}^m g_j Z_j$, where $g_j\in {{\mathbb {C}}}\llbracket Z_1,\ldots ,Z_m \rrbracket$. By the surjectivity of ${\mathfrak {b}}$ we can find $v_j\in {\mathfrak {S}}_0$ such that ${\mathfrak {b}}(v_j)=g_j$, $j=1,\ldots , m$. Then ${\mathfrak {b}}\left( u-\sum _{j=1}^mv_jZ_j\right) = {\mathfrak {b}}(u) - \sum _{j=1}^mg_jZ_j=0$.

We also have:

### Lemma 9.2

Assume that condition ($\mathcal {B}$) holds and also that ${\mathcal {V}}$ is minimal at 0. Then

$$\begin{aligned} \ker {\mathfrak {b}}= \bigcap _{k=1}^\infty \langle Z_m^k \rangle . \end{aligned}$$

Before we embark in the proof of Lemma 9.2 we show how it leads to the proof of Theorem 9.1. Indeed let $W_1,\ldots ,W_m$ be as in its statement. By Lemmas 9.1 and 9.2 we can write

$$\begin{aligned} W_k = \sum _{r=1}^m \gamma _{kr}Z_r, \quad \gamma _{kr} \in {\mathfrak {S}}_0. \end{aligned}$$

Since $Z_k(0)=0$ for every *k* we have

$$\begin{aligned} \hbox {d}W_k(0) = \sum _{r=1}^m \gamma _{kr}(0)\hbox {d}Z_r(0) \end{aligned}$$

and hence the matrix $(\gamma _{kr}(0))_{1\le k,r\le m}$ is invertible. By continuity it follows that the matrix of germs $(\gamma _{kr})_{1\le k,r\le m}$ is invertible and that its inverse $(\gamma ^{kr})_{1\le k,r\le m}$ is such that $\gamma ^{kr}$ belongs to ${\mathfrak {S}}_0$, since the latter is a ring. Furthermore we have

$$\begin{aligned} Z_k = \sum _{r=1}^m \gamma ^{kr}W_r \end{aligned}$$

and this concludes the proof of Theorem 9.1.

### Proof of Lemma 9.2

Let $V\subset U$ be an open neighborhood of the origin and let $u\in {\mathfrak {S}}(V)$ vanish to infinite order at 0. Assume first that ${\mathcal {V}}$ is minimal at the origin. By [10] there are an open set ${{\mathcal {U}}}$ in ${{\mathbb {C}}}^m$, a compact neighborhood of the origin $K\subset V$ (both indeed independent of *u*) and $h\in {\mathcal {O}}({{\mathcal {U}}})$ such that the following is true:

- $Z(K)\subset \bar{{\mathcal {U}}}$, for every $\alpha \in {{\mathbb {Z}}}^m_+$ the holomorphic function $\partial ^\alpha h$ extends continuously up to ${{\mathcal {U}}}\cup Z(K)$ and
  9.1 $$\begin{aligned} (\partial ^\alpha h) \circ Z= {\mathrm {M}}^\alpha u \hbox { on }K. \end{aligned}$$

$$\square $$

Notice in particular that if we consider the continuous functions on ${{\mathcal {U}}}\times {{\mathcal {U}}}$

$$\begin{aligned} U_\alpha (z,w) = |\partial ^\alpha h(z) - \sum _{|\beta |\le k-|\alpha |}\partial ^{\alpha + \beta }h(w)(z-w)^\beta /\beta !|/|z-w|^{|\alpha - k|}, \end{aligned}$$

defined as zero when $z=w$, they all extend continuously to $Z(K)\times Z(K)$. Consequently the family $\{u_{\alpha ,\beta }\}_{(\alpha ,\beta )\in {{\mathbb {Z}}}^m_+\times {{\mathbb {Z}}}^m_+}$, defined as

$$\begin{aligned} u_{\alpha ,\beta }(z,{\bar{z}}) = \left\{ \begin{array}{rl} (\partial ^\alpha h)|_{Z(K)} &{} \hbox { if}\ \beta =0,\\ 0 &{} \hbox { if}\ \beta \ne 0 \end{array}\right. \end{aligned}$$

is a Whitney family on *Z*(*K*).

By the Whitney extension theorem ([8], Theorem 2.3.6) for every *p* there is $H_p\in C^p({{\mathbb {C}}}^m)$ such that

$$\begin{aligned} (\partial _z^\alpha \partial ^\beta _{{\bar{z}}}H_p)|_{Z(K)} = \left\{ \begin{array}{rl} (\partial _z^\alpha h)|_K&{} \hbox { if}\ \beta =0,\\ 0 &{} \hbox { if}\ \beta \ne 0 \end{array}\right. \end{aligned}$$

and $|\alpha |+|\beta |\le p$. By (9.1) all the derivatives of $H_{p}$ of order $\le p-1$ vanish at the origin and hence we must have $|H_p(z)| = {\mathrm {O}}(|z|^{p})$, for *z* near the origin in ${{\mathbb {C}}}^m$. In particular

$$\begin{aligned} |u(x,t)| = |h(Z(x,t))| = |H_p(Z(x,t))| = {\mathrm {O}}(|Z(x,t)|^p) . \end{aligned}$$

Hence, by (${\mathfrak {B}}$), we obtain

9.2 $$\begin{aligned} |u(x,t)|= {\mathrm {O}}(|Z_m(x,t)|^p),\quad p\ge 0. \end{aligned}$$

Repeating the argument with ${\mathrm {M}}^\alpha u$ replacing *u* we further obtain

9.3 $$\begin{aligned} |({\mathrm {M}}^\alpha u)(x,t)|= {\mathrm {O}}(|Z_m(x,t)|^p),\quad p\ge 0. \end{aligned}$$

Define $v_k(x,t)=u(x,t)/Z_m^k(x,t)$, if $Z_m(x,t)\ne 0$, $v_k(x,t)=0$ when $Z_m(x,t)=0$. Then (9.2) implies that $v_k(x,t)$ is continuous and is smooth when $x_m\ne 0$. By a standard result in distribution theory ([8], Theorem 3.1.3) we have ${\mathrm {L}}_j v_k=0$ and

9.4 $$\begin{aligned} {\mathrm {M}}_\ell v_k = ({\mathrm {M}}_\ell u)/Z_m^k -k u/Z_m^{k+1} \end{aligned}$$

in the distribution sense, $j=1,\ldots ,n$, $\ell =1,\ldots ,m$. By (9.3) it follows that the right hand side of (9.4) is continuous (if defined as zero when $Z_m=0$) and then by ([8], Theorem 3.1.7) it follows that $v_k\in C^1$ and that ${\mathrm {L}}_j v_k=0$ in the classical sense, $j=1,\ldots ,n$. If we iterate the argument it follows that $v_k$ is smooth for every $k\in {{\mathbb {Z}}}_+$ and also that ${\mathrm {L}}_j v_k=0$ for all $j=1,\dots ,n$ and all $k\in {{\mathbb {Z}}}_+$.

Next we assume that $Z_1,\ldots ,Z_m$ are real-analytic functions and let *V* be an open neighborhood of the origin in *U*. By the Baouendi–Treves approximation theorem the following can be said: there is an open ball $W\subset \subset V$ centered the origin such that every element in ${\mathfrak {S}}(V)$ is constant on the set

$$\begin{aligned} F_0 = \{ (x,t)\in W: Z(x,t)=0\}. \end{aligned}$$

Let $u\in {\mathfrak {S}}(V)$ vanish to infinite order at the origin. Then ${\mathrm {M}}^\alpha u\in {\mathfrak {S}}(V)$ ($\alpha \in {{\mathbb {Z}}}^m_+$) vanish at the origin and consequently vanish on $F_0$. Consequently all derivatives of *u* vanish on $F_0$ and hence Taylor’s formula gives, for every $q\in {\mathbb {Z}}_+$,

$$\begin{aligned} |u(x,t)|\le A_q |(x,t)-(x',t')|^q,\quad (x,t)\in W, \, (x',t')\in F_0, \end{aligned}$$

where $A_q$ only depends on bounds for the derivatives of *u* on ${\bar{W}}$ of order *q*. Taking the infimum over $(x', t')\in F_0$ we obtain

$$\begin{aligned} |u(x,t)|\le A_q \hbox {dist}((x,t),F_0)^q,\quad (x,t)\in W. \end{aligned}$$

Let $K\subset W$ be a compact neighborhood of the origin. Since $F_0$ is the zero set of the real-analytic function $f\doteq |Z_1|^2+\ldots +|Z_m|^2$ by Lojasiewicz inequality (cf. [M], Theorem 4.1) there are constants $C>0$ and $\gamma >0$ such that

$$\begin{aligned} \hbox {dist}((x,t),F_0)^\gamma \le C|Z(x,t)|^2, \quad (x,t)\in K. \end{aligned}$$

Hence

$$\begin{aligned} |u(x,t)|\le C^{1/\gamma }A_q|Z(x,t)|^{2q/\gamma },\quad (x,t)\in K, \end{aligned}$$

for every $q\in {\mathbb {Z}}_+$. Again by (${\mathfrak {B}}$) we derive the validity of (9.2) in this case and the preceding argument applies without modifications. The proof of Lemma 9.2 is complete. $\square$

### Corollary 9.1

Assume that ${\mathcal {V}}$ is a real analytic locally integrable structure of rank $N-1$ (that is, ${\mathcal {V}}^\perp$ is a complex fiber subbundle of ${{\mathbb {C}}}{\mathrm {T}}^*\varOmega$). Then the conclusion of Theorem 8.1 holds at every point in $\varOmega$.

Indeed when the rank of ${\mathcal {V}}$ is $N-1$ and $p\in \varOmega$ then ${\mathcal {V}}$ is not hypocomplex at *p* if and only if property (${\mathfrak {B}}$) holds at *p* ([3], Corollary 6.2).

**C.** Besides the hypocomplex case, the conclusion of Theorem 9.1 holds in some cases when it is not known whether condition (${\mathfrak {B}}$) is valid or not. As in section 6(A) we assume that ${\mathcal {V}}$ is the locally integrable structure associated to a smooth, minimal, (weakly) convex hypersurface $\varOmega \subset {{\mathbb {C}}}^m$. Assume $0\in \varOmega$. We claim that

$$\begin{aligned} {\mathfrak {m}}= \langle z_1|_\varOmega ,\ldots ,z_m|_\varOmega \rangle . \end{aligned}$$

Indeed let *V* be an open neighborhood of the origin in $\varOmega$ and let $f\in {\mathfrak {S}}(V)$ satisfy $f(0)=0$. Then there is a weakly convex smooth domain ${{\mathcal {U}}}$ in ${{\mathbb {C}}}^m$ such that $\partial {{\mathcal {U}}}\cap \varOmega \doteq W\subset V$ is an open neighborhood of the origin in $\varOmega$ and there is $F\in {\mathcal {O}}({{\mathcal {U}}})\cap C^\infty (\overline{{\mathcal {U}}})$ such that $F=f$ in *W*. Since $F(0)=0$ we can write, for $z\in \overline{{\mathcal {U}}}$,

$$\begin{aligned} F(z) = F(z_1,\ldots , z_n)= \int _{0}^1 \frac{\partial }{\partial t}(F(tz_1,\ldots , tz_n))dt \end{aligned}$$

where the integral is well-defined because, by convexity, $(tz_1,\ldots ,tz_n)\in \overline{{\mathcal {U}}}$ for $0\le t\le 1$. By the chain rule we get

$$\begin{aligned} F(z) = \sum _{j=1}^m \int _0^1 z_j \frac{\partial F}{\partial z_j}(tz_1,\ldots ,tz_m)dt= \sum _{j=1}^m z_j F_j(z), \end{aligned}$$

where

$$\begin{aligned} F_j(z)\doteq \int _0^1\frac{\partial F}{\partial z_j}(tz_1,\ldots ,tz_n)dt \end{aligned}$$

is holomorphic on ${{\mathcal {U}}}$ and smooth up to the boundary for all $1\le j\le m$, so that

$$\begin{aligned} f=\sum _j (F_j|_W) (z_j|_W) . \end{aligned}$$

Such argument applies for instance to the hypersurface

$$\begin{aligned} \varOmega _\sharp = \{(z,w)\in {{\mathbb {C}}}^2:\, {{\mathsf {Im}}\,}w = e^{-1/|z|}\} \end{aligned}$$

which is convex, minimal but not of finite type. Note that we do not currently know whether the Borel property holds for the CR structure induced on $\varOmega _\sharp$.

## Principal manifold ideals

We continue to work under the notation established in the last section. Let $f_1,\ldots , f_\ell \in {\mathfrak {m}}$ and consider the ideal $I=\langle f_1,\ldots , f_\ell \rangle \subset {\mathfrak {m}}$. We say that *I* is a *manifold ideal* if

10.1 $$\begin{aligned} \left( \hbox {d}f_1\wedge \ldots \wedge \hbox {d}f_\ell \wedge \hbox {d}\overline{f_1}\wedge \ldots \wedge \hbox {d}\overline{f_\ell }\right) (0)\ne 0. \end{aligned}$$

We denote by *V*(*I*) the germ $\{f_1=\ldots =f_\ell =0\}$, and call it the *variety* of *I*.

### Lemma 10.1

If *I* is a manifold ideal then *V*(*I*) is the germ of a regular submanifold of real codimension $2\ell$ of ${\mathbb {R}}^N$ around 0. Moreover, we can find a coordinate system $(x_1,\ldots ,x_m,t_1,\ldots , t_n)$ centered at the origin in ${{\mathbb {R}}}^N$ and solutions $Z_1,\ldots ,Z_m$ satisfying the properties listed in Section 1B such that $I=\langle Z_1,\ldots ,Z_\ell \rangle$.

### Proof

The first claim is an immediate consequence of (10.1) whereas the second follows from the arguments in ([4], Theorem I.10.1) as done in ([3], Section 4).

We shall now restrict our attention * principal maximal ideals*, that is the ones generated by a single element $f\in {\mathfrak {S}}_0$ such that $(df\wedge \hbox {d}\overline{f})(0)\ne 0$. For any submanifold germ *V* of ${\mathbb {R}}^N$ around 0, we denote by ${{\mathcal {I}}}(V)$ the *ideal* of *V*, i.e. the ideal of ${\mathfrak {S}}_0$ consisting of those germs vanishing on *V*. It is clear that $I\subset {{\mathcal {I}}}(V(I))$. Our aim is to show that the opposite inclusion also holds:

### Theorem 10.1

Let $I\subset {\mathfrak {S}}_0$ be a principal manifold ideal. Then ${{\mathcal {I}}}(V(I))=I$.

We remark that in the previous statement no assumption is made about the minimality of ${\mathcal {V}}$ nor on the validity of property (${\mathfrak {B}}$). In order to prove Theorem 10.1 we first prove a simple lemma.

### Lemma 10.2

Let $k\ge 2$. Then the function ${{\mathbb {C}}}\ni z\rightarrow \phi _k(z)={\overline{z}}^k/z\in {{\mathbb {C}}}$ is of class $C^{k-2}$.

### Proof

Clearly $\phi _k$ extends continuously to 0 since the function ${\overline{z}}/z$ is bounded. Choose $j,\ell \in {\mathbb {N}}$ such that $j+\ell \le k-2$. Then

$$\begin{aligned} \frac{\partial ^{j+\ell }}{\partial z^j\partial {\overline{z}}^\ell }\phi _k(z)= (-1)^j\frac{j!k!}{\ell !} \frac{{\overline{z}}^{k-\ell }}{z^{j+1}} = (-1)^j\frac{j!k!}{\ell !} \left( \frac{{\overline{z}}}{z}\right) ^{j+1} {\overline{z}}^{k-(j+\ell +1)} \end{aligned}$$

is again continuous around 0 by the boundedness of ${\overline{z}}/z$, since $k-(j+\ell +1)\ge 1$.

### Proof of Theorem 10.1

We can assume that we are in the situation described in Section 1B in such a way $I=\langle Z_1 \rangle$ (cf. Lemma 10.1). Moreover since *I* is a principal maximal ideal we can even assume that $\phi _1(x,t)=t_1$.

Let $g\in {\mathfrak {S}}_0$ vanish on $V(I)=\{Z_1=0\}=\{x_1=t_1=0\}$. Our goal is to show that $g=g_\bullet Z_1$ for some $g_\bullet \in {\mathfrak {S}}_0$.

We start by setting

10.2 $$\begin{aligned} h_k = \sum _{j=1}^k \frac{(-1)^j}{j!} Z_1^j\, {\mathrm {M}}_1^j g, \quad k\ge 1. \end{aligned}$$

Notice that $h_k\in {\mathfrak {S}}_0$. We claim that

10.3 $$\begin{aligned} {\mathrm {M}}^\alpha (g+ h_k)|_{V(I)} = 0,\quad \alpha \in {\mathbb {Z}}^m_+,\,\, |\alpha |\le k. \end{aligned}$$

In order to prove (10.3) we first note that if $j\ge 2$ then $0={\mathrm {M}}_j Z_1= {\mathrm {M}}_j x_1$ and hence ${\mathrm {M}}_j$ only involves $\partial /\partial x_2,\ldots , \partial /\partial x_m$. Thus if $v\in C^\infty _0$ vanishes on *V*(*I*) the same is true for ${\mathrm {M}}^\alpha v$ if $\alpha =(0,\alpha _2,\ldots ,\alpha _m)\in {\mathbb {Z}}^m_+$. Thus (10.3) follows if we show that ${\mathrm {M}}_1^\ell (g+h_k)=0$ on *V*(*I*) if $\ell \le k$. By Leibniz rule we have

$$\begin{aligned} {\mathrm {M}}_1^\ell (g+ h_k)= & {} {\mathrm {M}}_1^\ell g + {\mathrm {M}}_1^{\ell }\left\{ \sum _{j=1}^k \frac{(-1)^j}{j!} Z_1^j {\mathrm {M}}_1^j g\right\} \\= & {} {\mathrm {M}}_1^\ell g + \sum _{j=1}^k \sum _{r=0}^{\min \{j,\ell \}}\frac{(-1)^j}{j!}\left( {\begin{array}{c}\ell \\ r\end{array}}\right) \frac{j!}{(j-r)!}Z_1^{j-r} {\mathrm {M}}_1^{j+\ell -r} g \end{aligned}$$

If we restrict this last sum to *V*(*I*) and recall that $\ell \le k$ we obtain

$$\begin{aligned} \sum _{j=1}^\ell (-1)^j\left( {\begin{array}{c}\ell \\ j\end{array}}\right) ({\mathrm {M}}^\ell g)|_{Z_1=0} = - ({\mathrm {M}}^\ell g)|_{Z_1=0}\, , \end{aligned}$$

which completes the proof of (10.3).

Let $g' = g/Z_1$. Then $g'$ is defined – and is a solution of ${\mathcal {V}}$ – on the complement of $\{Z_1=0\}$. It is enough to prove that for any $k\ge 2$ the germ $g'$ extends across $\{Z_1=0\}$ as a function of class $C^{k-2}$. If $h_{k-1}$ is as in (10.2) then $h_{k-1}/Z_1\in {\mathfrak {S}}_0$ and hence we are left to showing that $(g+h_{k-1})/Z_1$ extends accross $Z_1=0$ as a function of class $C^{k-2}$.

We take advantage of (10.3). By Taylor’s formula we can write

$$\begin{aligned} g+h_{k-1} = A_k \, Z_1 + B_k \overline{Z_1}^k , \end{aligned}$$

where $A_k,B_k\in C^\infty _0$. Consequently by Lemma 10.2 we can write

$$\begin{aligned} (g+h_{k-1})/Z_1 = A_k + B_k\, \phi _k(Z_1) \end{aligned}$$

is of class $C^{k-2}$, which completes the proof. $\square$

### Example 1

If the assumption that *I* is a manifold ideal is not satisfied, the conclusion of Theorem 10.1 can fail to hold. For instance, let ${\mathcal {V}}$ be the locally integrable structure on ${{\mathbb {R}}}^3$, with coordinates written as (*x*, *y*, *s*), whose orthogonal ${\mathcal {V}}^\perp$ is spanned by the differential of the functions

$$\begin{aligned} Z=x+iy, \ \ W = s + i(x^2 + y^2) \end{aligned}$$

(this is the standard Hans Lewy structucture on ${{\mathbb {C}}}^2$), and define $I=\langle W\rangle$. Then *I* is not a manifold ideal, and we have that $V(I) = \{0\}$ and ${\mathcal {I}}(V(I))=\langle Z,W \rangle ={\mathfrak {m}}\supsetneq I$. Also note that ${\mathfrak {m}}$ does not coincide with the radical of the ideal *I*, since there is no $k\in {\mathbb {Z}}_+$ such that $Z^k/W$ is of class $C^\infty$ around 0. It follows that the *Nullstellensatz* does not hold for a (general) ideal of ${\mathfrak {S}}_0$.

### Example 2

On the other hand, consider the structure ${\mathcal {V}}$ on ${{\mathbb {R}}}^5$, with coordinates written as $(x_1,x_2,y_1,y_2,s)$ whose orthogonal ${\mathcal {V}}^\perp$ is spanned by the differential of the functions

$$\begin{aligned} Z_1=x_1+iy_1, \ \ Z_2 = x_2 + iy_2, \ \ W = s + i(x_1^2 + y_1^2 - x_2^2 - y_2^2). \end{aligned}$$

Once again we have that $I=\langle W\rangle$ is not a manifold ideal, but in this case we have ${{\mathcal {I}}}(V(I)) = I$. Indeed it is well known that ${\mathcal {V}}$ is hypocomplex at the origin [1]. On the other hand, writing the complex coordinates in ${{\mathbb {C}}}^3$ as $(z_1,z_2,w)$ we see that that if $H\in {\mathcal {O}}^{(3)}$ vanishes on *V*(*I*) then $H(z_1,z_2,0)$ vanishes on $|z_1|^2=|z_2|^2$, and consequently $H=wH_1$, with $H_1\in {\mathcal {O}}^{(3)}$. This proves our claim.

## Some open questions

A certain number of questions arise, in our opinion, naturally from the results presented in the previous sections. Despite the quite elementary nature of some of them (the topic of the algebraic properties of the ring ${\mathfrak {S}}_p$ appears to be to some extent unexplored) their treatment seems to lead to delicate analytic issues. The following is an (incomplete) list of the problems which are for us most natural and interesting:

### Question 1

Is the necessary condition found in Theorem 6.1 also sufficient for the surjectivity of the Borel map?

We conjecture that this should be the case, at least when the structure ${\mathcal {V}}$ is real-analytic.

### Question 2

Does the conclusion of Theorem 10.1 hold for a non principal manifold ideal?

The method used in the proof of Theorem 10.1 does not extend easily to ideals generated by more than one solution.

### Question 3

Is there an example in which the maximal ideal ${\mathfrak {m}}$ is *not* generated by the basic solutions $Z_1,\ldots ,Z_m$?

The results in Sect. 9 show that this property in various situations, far apart from each other. The knowledge of the behavior of the Borel map seems to be important in most of the proofs, with the exception of the argument in Sect. 9C.

### Question 4

For what values of *p* and *q* does the structure in Theorem 8.3 satisfy the Borel property?

We expect that the Borel property should hold precisely when *p* and *q* have different parity.

### Question 5

Is the image of ${\mathfrak {b}}$ always a suitable quotient of a ring of the form $({{\mathbb {C}}}\{Z_1,\ldots ,Z_p\})\llbracket Z_{p+1},\ldots ,Z_m\rrbracket$?

In other words, in the cases settled so far the image of ${\mathfrak {b}}$ consists of formal series in a subset of variables whose coefficients are holomorphic functions in the other variables (more precisely, these coefficients must have a common radius of convergence).

### Question 6

Suppose that two integrable structures ${\mathcal {V}}_1, {\mathcal {V}}_2$ are not hypocomplex (e.g. correspond to pseudoconvex hypersurfaces $M_1,M_2\subset {\mathbb {C}}^n$), and the solutions rings ${\mathfrak {S}}_0^1$, ${\mathfrak {S}}_0^2$ are isomorphic. Does it follow that ${\mathcal {V}}_1,{\mathcal {V}}_2$ are locally equivalent (i.e. that $M_1$ and $M_2$ are locally CR diffeomorphic)?

It is clear that the answer to the previous question is negative if ${\mathcal {V}}_1,{\mathcal {V}}_2$ are hypocomplex, since both rings of solutions will always be isomorphic to the ring of convergent power series in *n* variables: if, however, there are enough solutions, one might hope that the ring ${\mathfrak {S}}_0$ contains enough information.

## Footnotes

1 Here *d* is any distance function defined near *p* by using local coordinates. It is easily seen that the definition of the ideals ${\mathfrak {m}}^k_p$ is invariant.
